# *In vitro* profiling of *Trypanosoma cruzi* inhibitors identified from High throughput Screening and application to parasite painting

**DOI:** 10.1016/j.ijpddr.2026.100651

**Published:** 2026-05-14

**Authors:** Kyung-Hwa Baek, Hyeryon Lee, Sooyoung Byun, Olga Genilloud, Jean-Robert Ioset, David Shum, Joo Hwan No

**Affiliations:** a Institut Pasteur Korea, 16 Daewangangyo-ro 712 beon-gil, Bundang-gu, Seongnam-si, Gyenonggi-do, 13488, Republic of Korea; b Fundación MEDINA, Avda. del Conocimiento 34, Armilla, 18016, Granada, Spain; c Drugs for Neglected Diseases Initiative, 15 Chemin Camille-Vidart, 1202, Geneva, Switzerland; d Department of Advanced Drug Discovery & Development, Institut Pasteur Korea School, University of Science and Technology (UST), 16 Daewangangyo-ro 712 beon-gil, Bundang-gu, Seongnam-si, Gyenonggi-do, 13488, Republic of Korea

**Keywords:** *Trypanosoma cruzi*, High throughput screening, Inhibitors, Phenotypic profiling, Parasite painting

## Abstract

Chagas disease, a neglected tropical disease caused by *Trypanosoma cruzi*, urgently requires next-generation therapeutics due to the limitations of use of benznidazole and nifurtimox, including adverse effects, long treatment period and still unproven efficacy in chronic cases. With limited knowledge of *T. cruzi* biology regarding validated targets that could be addressed from a drug discovery perspective, most drug discovery efforts associated to the identification of new *T. cruzi* active chemotypes has been relying upon cell-based assays. The failure of repurposing the CYP51 acting antifungal drug posaconazole through clinical trials has highlighted the need for a more sophisticated characterization of phenotypically identified *T. cruzi* inhibitors via more stringent triage compounds before engaging with downstream drug development. In this study, we have evaluated 2422 compounds against the intracellular amastigotes of *T. cruzi* to identify novel starting points for drug development. The 30 identified inhibitors of *T. cruzi* growth including 5 serotonin-dopamine receptor antagonists and 7 TGF-β receptor inhibitors were then used as chemical tools to profile the drug action effects in terms of *T. cruzi* amastigote quantification measurements, kinetics of action, susceptibility in different strains as well as forms of the parasite. In terms of kinetics of action, fast-acting compounds inhibited the proliferation of intracellular amastigotes within 48 h of incubation, whereas slow-acting compounds required prolonged exposure to achieve comparable growth inhibition. Although most compounds were active against both Y and Dm28c strains, the correlation between their IC_50_ values was only moderate. Among the 30 identified inhibitors, 6 displayed potent inhibitory activity against all three parasite life-cycle stages—amastigotes, trypomastigotes, and epimastigotes. Lastly, the “parasite painting” methodology was applied to trypomastigotes of the parasite to classify compounds based on morphological perturbations. The series of assays applied in this study offer tools to characterize and prioritize inhibitors for the downstream discovery process.

## Highlights

• HTS of 2422 compounds identified 30 selective inhibitors of *T. cruzi* amastigote.
• Distinct readouts and times revealed differences in potency and killing kinetics.
• Strain- and stage-specific susceptibility varied across *T. cruzi* profiles.
• “Parasite-painting” clustered trypomastigote inhibitors by morphology and class.
• Two Serotonin–dopamine antagonists showed multi-stage activity against *T. cruzi.*

## Graphical abstract

**Figure:**
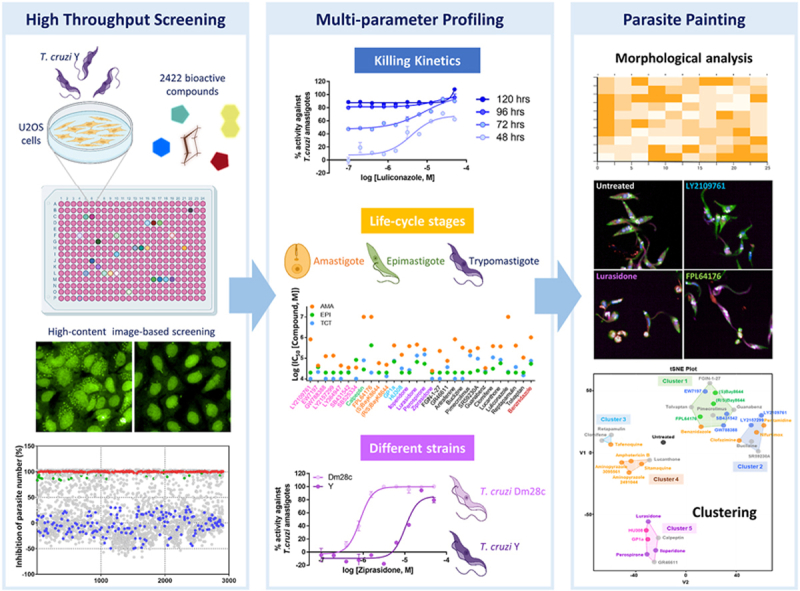

## 1 Introduction

Chagas disease (CD) is an infectious disease caused by the protozoan parasite *Trypanosoma cruzi*. Since its first identification by Carlos Chagas in Brazil in 1909, the disease has become endemic in Central and Latin America, with 70 million people at risk (Lidani et al., 2019; PAHO, 2025). Currently, an estimated 6 to 7 million individuals are infected, leading to approximately 12,000 deaths annually (WHO, 2025). In Brazil alone, a mortality rate of 2.78 deaths per 100,000 people and 5000 annual deaths is reported (da Silva et al., 2025). Due to active human migrations, CD patients have been frequently reported in North America, Europe, Australia, and Japan, becoming a worldwide health problem (Lidani et al., 2019).

The disease is transmitted by blood-sucking triatomine bugs, also known as “kissing bugs” and the parasite cycles through three main forms: epimastigotes (the proliferative form in the vector), amastigotes (the proliferative form in vertebrate host cells), and trypomastigotes (the infective form in the vertebrate host) (Rassi et al., 2010). CD represents complex pathophysiology with variable clinical manifestations. Once infected by *T. cruzi*, patients first experience an acute phase characterized by a high level of parasitemia (Barrett et al., 2003). Oftentimes asymptomatic, symptomatic cases can be accompanied by prolonged fever, myalgia, lymphadenitis, hepato-, and splenomegaly, followed by relief of symptoms without drug treatment as the disease enters the chronic phase (Barrett et al., 2003). In the chronic phase, 60 to 70% of patients live with silent symptoms, but the remaining 30 to 40% develop cardio or digestive clinical manifestations, which can lead to death if left untreated (Rassi et al., 2010). There are two drugs, belonging to the same class of nitroheterocyclics, available for treating patients with CD: benznidazole (Radanil®, Roche, introduced in 1971) and nifurtimox (Lampit®, Bayer), launched more than 50 years ago (Coura and De Castro, 2002). Both drugs are effective in the acute phase of the disease, but a high proportion (∼70%) of patients suffer from adverse effects during the prolonged course of treatment that frequently leads to treatment discontination (Marin-Neto et al., 2007; Molina et al., 2015). Although there is no consensus on the effect of benznidazole in the chronic phase, studies have shown that drug treatment was able to prevent the onset or delay the progression of the disease (Bern et al., 2007; Fabbro et al., 2007) However, in the BENEFIT clinical study (Benznidazole Evaluation for Interrupting Trypanosomiasis), involving patients with chronic chagasic cardiomyopathy, the most frequent and severe manifestation of CD, showed no delay in disease progression with benznidazole treatment (Marin-Neto et al., 2008).

Due to the adverse effects and varying levels of efficacy of current drugs, there is an urgent need to develop more safe and effective treatments for CD. An antifungal drug, posaconazole, approved for human use, was initially found to be associated with quite promising results both *in vitro* and *in vivo*. The compound showed a 90% and 60% cure rate in the mouse model of acute and chronic CD, respectively (Francisco et al., 2015, 2016). Based on the promising preclinical results, two clinical trials were conducted (NCT01162967 and NCT01377480). In both trials, posaconazole was shown to successfully eliminate *T. cruzi* from circulation, however, the effect was rather trypanostatic, as parasite suppression was not sustained in long-term evaluation after the discontinuation of treatment (Morillo et al., 2017). Another azole-type compound, E1224 aka fosravuconazole, the prodrug form of ravuconazole subsequently investigated in phase II clinical trials led to similar outcome as parasite relapse was observed a year after the end of treatment (Francisco et al., 2015; Torrico et al., 2018).

As part of the effort to generate more efficacious clinical candidates for CD, cell-based phenotypic screening methods have been widely utilized. The epimastigote form of *T. cruzi* can be readily obtained in abundance from axenic culture and used for the screening of small molecules through a variety of detection methods (Muelas-Serrano et al., 2000; Vega et al., 2005; Rolón et al., 2006). However, epimastigotes are the form in the insect vector, which does not reflect the infection in humans. To test compounds in more physiologically relevant conditions, *in vitro* culture of extracellular trypomastigotes and intracellular amastigotes with engineered *T. cruzi* with reporters such as the β-galactosidase gene (lacZ), firefly luciferase (luc), or fluorescent proteins were utilized for screening compounds (Buckner et al., 1996; Bettiol et al., 2009; Canavaci et al., 2010). More recently, the advancement of high-content imaging systems has enabled high-throughput screening of compounds against the intracellular amastigote form of *T. cruzi* in a more sophisticated fashion. The images are analyzed in a way that allows to quantify the total number of parasites and host cells, as well as the ratio of infection of host cells present in the wells. The measurement of host cell cytotoxicity is then used to identify compounds that selectively kill the *T. cruzi* parasite (Engel et al., 2010; Bustamante and Tarleton, 2011; De Muylder et al., 2011; Sykes and Avery, 2013; Moon et al., 2014; Alonso-Padilla et al., 2015). With the emergence of these high-throughput screening technologies, small to large-sized chemical libraries were screened to identify starting points for anti-trypanosomal drug discovery, some of which have progressed to the later stages of development (Andriani et al., 2011; Dandapani et al., 2014; Annang et al., 2015; Jeffrey Neitz et al., 2015; Peña et al., 2015; Sykes and Avery, 2015; Bernatchez et al., 2020). However, the global Chagas R&D pipeline remains quite thin, with LXE408 and oxaboroles (AN2-502998 and DNDi-6148) being to most advanced candidates that have entered or are being considered to enter clinical stage (AN2, 2025; Novatis, 2025) (https://dndi.org/research-development/portfolio/; last visited 23. Feb 2026).

As part of the effort to identify new anti-*T. cruzi* pharmacophores and understand the characteristics of activities under different conditions of *in vitro* assays, in this study, we have screened 2422 compounds to discover a set of 30 inhibitors and analyze their activities in various terms including quantification measures, compound incubation kinetics, and parasite form. Additionally, a classification of compounds was performed based on the morphological profiling of inhibitor-perturbed parasites.

## 2 Materials and methods

### 2.1 Reagents and mammalian cell cultures

Benznidazole (Carbosynth, Berkshire, UK) was prepared in DMSO and used as a reference drug. FDA-approved drugs were purchased from MedChemExpress (Monmouth Junction, NJ, USA), Selleckchem (Houston, TX, USA), Sigma-Aldrich (Saint Louis, MO, USA), or Tocris (Bristol, UK), and prepared as 5 mM stock in DMSO. The final concentrations of DMSO were below 0.5% and 1% in *in vitro* assays, respectively. LLC-MK2 monkey kidney cells and U2OS human osteosarcoma cells were maintained in Dulbecco's modified Eagle's medium (DMEM, Welgene, Gyeongsan-si, Republic of Korea) medium supplemented with high glucose, 10% FBS, and 1% penicillin/streptomycin (P/S) at 37 °C with 5% CO_2_ in air (Yang et al., 2017).

### 2.2 In vitro activity test against *T. cruzi* amastigotes and HTS

U2OS cells were seeded at 0.8 × 10^4^ cells per well in a 384-well culture plate (Greiner Bio-One, Kremsmünster, Austria) containing 0.5% DMSO (negative control), benznidazole (at 400 μM, positive control), and screening compounds (at 10 μM) in low glucose DMEM medium supplemented with 2% fetal bovine serum (FBS, BD Difco™, Franklin Lakes, NJ, USA) at 37 °C in the presence of 5% CO_2_. Simultaneously, trypomastigotes of *T. cruzi* Y were added to the cells at a parasite-to-cell ratio of 4:1. After 72 h, the cells and parasites were fixed with 4% paraformaldehyde (PFA), washed with DPBS, and stained using 5 μM DRAQ5™ (Thermo Fisher, Rockford, IL, USA) (Siqueira-Neto et al., 2012; Yang et al., 2015). The images were acquired using an Operetta (PerkinElmer Inc., Waltham, MA, USA) with a 20× air objective. The images were further analyzed using Columbus software (PerkinElmer Inc., Waltham, MA, USA) to quantify parasite numbers, host cell numbers, and infection ratios. Host cell nuclei were first detected using DRAQ5™ signal, and cell boundaries were masked using low-intensity cytosolic signals (Baek et al., 2020). Parasites were identified by small-sized DRAQ5™ nuclear signals within the masked host cell areas. The infection ratio (IR) was calculated as the number of infected cells divided by the total number of cells, and the average number of parasites per cell (PAR) was determined as the total number of parasites divided by the number of infected cells. Compounds selected from the screen were further assessed in a dose-dilution manner (two-fold serial dilution for 10 points starting from 100 μM) to derive IC_50_ values.

### 2.3 In vitro activity test against *T. cruzi* trypomastigotes and epimastigotes

For the tissue-culture derived trypomastigotes (TCTs) assay, LLC-MK2 cells maintained in DMEM with high glucose, 10% FBS, and 1% Penicillin/Streptomycin (P/S) at 37 °C with 5% CO_2_ in air were used as host cells to amplify either the Y or Dm28c strain. LLC-MK2 cells were infected with frozen stocks of TCTs from the Y or Dm28c strain in low glucose DMEM supplemented with 2% FBS and 1% P/S at 37 °C with 5% CO_2_ in air. TCTs were obtained from the supernatant 6∼7 days after infection by centrifugation at 2000×g for 10 min. Assays were performed in 384-well plates, seeded with *T. cruzi* trypomastigotes (10^6^ parasites per well). The trypomastigotes were treated with various drug concentrations or 0.5% DMSO (negative control) and incubated for 24 h at 37 °C with 5% CO_2_. Parasite viability was assayed by measuring resazurin reduction to resorufin. Resazurin sodium salt (200 μM, Sigma-Aldrich) was added, and the samples were incubated for 5 h. After incubation, the parasites were fixed using 4% PFA, and fluorescence was measured using a SpectraMax plate reader (San Jose, CA, USA) at 590 nm (emission) and 530 nm (excitation) (Rolón et al., 2006).

For the epimastigotes assay, *T. cruzi* epimastigotes were cultured in liver infusion tryptose (LIT) medium (BD Difco™, Franklin Lakes, NJ, USA) with 10% FBS at 28 °C. Exponentially growing epimastigotes were treated with various drug concentrations or 0.5% DMSO as a negative control and incubated for 3 days at 28 °C. Assays were performed in 384-well plates seeded with *T. cruzi* epimastigotes (10^6^ parasites per well). Growth inhibition was assessed using the resazurin assay, similar to the TCT assay.

### 2.4 *T. cruzi* trypomastigote painting assay

To fluorescently label parasites, tissue-culture derived trypomastigotes were plated in 384-well black plate in duplicates, exposed to IC_50_ values of compounds for 24 h and stained with Lysotracker Red DND-99 (Invitrogen™ L7528) and MitoTracker641 (Invitrogen™ M22426) for 30 min at RT. After fixation with 4% Paraformaldehyde, parasites were further stained with Hoechst 33342 (Invitrogen™ H3570) and WGA-Alexa488 (Invitrogen™ W11261) in 0.1 % TrixonX-100/DPBS for 4 h at RT. Stained parasites were diluted and imaged the Opera Phenix Plus high-content microscopes with a 63× water objective. Nine fields were captured per well at a single Z-plane as settled by image-based autofocus on four synchronized channels (Mirabelli et al., 2021).

### 2.5 Image processing and analysis

SimA (SignalsImageArtist) image analysis software (PerkinElmer Inc., Waltham, MA, USA) was used to segment organelles in parasites and extract 2817 features per parasite of images. Nuclei were identified by Hoechst33342 channel and used as criteria to set segmentation algorithm for detecting parasite boundaries from Alexa488 channel. These nuclei and cell boundaries were used to measure morphological features including shape, size, texture, and fluorescent intensity of four channels. Prior to analysis, features with more than 5% missing (NA) values were removed, and remaining NA values were imputed using median values. All remaining features were scaled, correlations were calculated, and highly correlated features (>0.85) were removed. Based on the mean average values of the well, t-distributed Stochastic Neighbor Embedding (t-SNE) was performed using Rtsne package, and compound clustering was visualized by ggplot2 in R (Hughes et al., 2020).

### 2.6 Statistical analysis

Values obtained from the image analysis algorithm were further analyzed using GraphPad Prism 6 (GraphPad Software, Boston, MA, USA) for graphical representations and determination of half-maximal inhibitory concentration (IC_50_) values. The assays were conducted in three independent experiments and values are expressed as mean ± SD.

## 3 Results

### 3.1 Identification of intracellular *T. cruzi* growth inhibitors through high-content screening

**Fig. 1:**
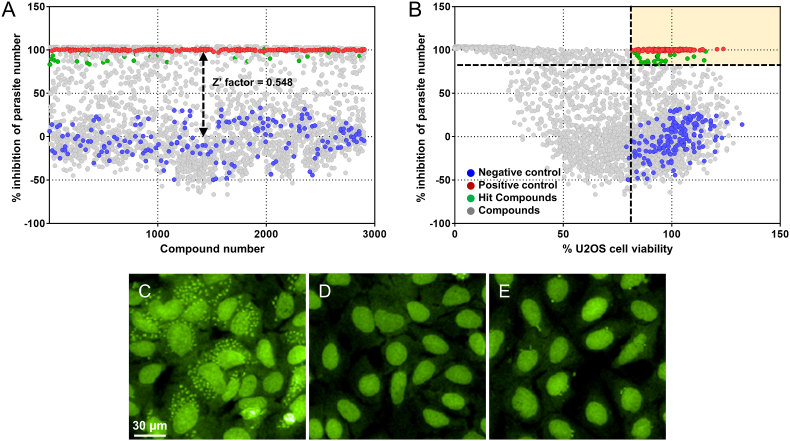
Screening of bioactive compounds against intracellular *T. cruzi* Y amastigote growth in U2OS cells. Results from high-throughput screening of 2422 compounds (gray), negative controls (blue, 0.5% DMSO), positive controls (red, benznidazole), and 30 selected hits (green) with >85% inhibition of parasite growth and >85% cell viability are shown (A, B). Representative images of 0.5% DMSO (C), 400 μM benznidazole (D), and LY2109761-treated samples after 72 h incubation. Scale bar = 30 μm.

**Fig. 2:**
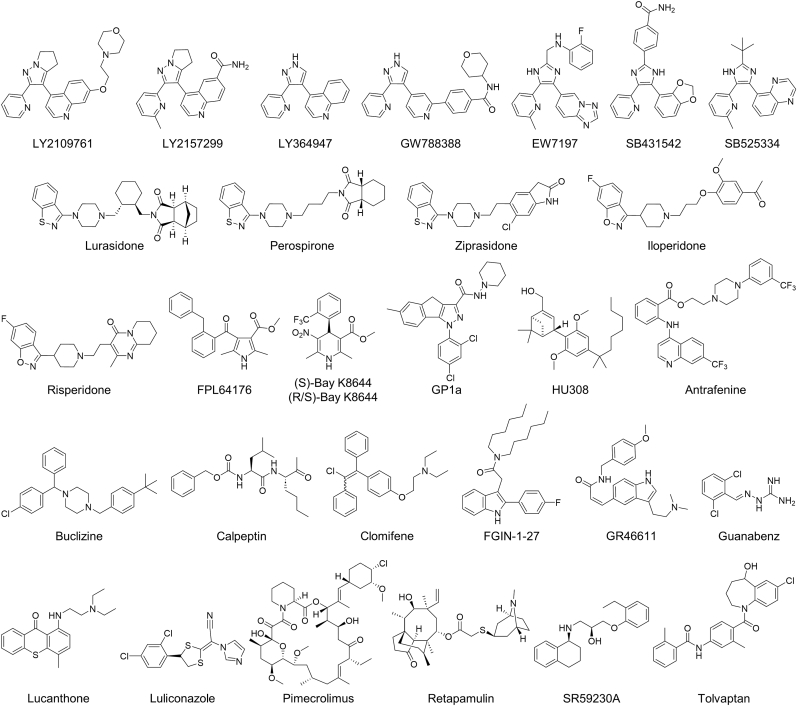
Structures of selected 30 hit compounds, including TGF-β receptor inhibitors (LY2109761, LY2157299, LY364947, GW788388, EW7197, SB431542, and SB525334), serotonin-dopamine antagonists (lurasidone, perospirone, ziprasidone, iloperidone, and risperidone), L-type Ca^2+^ channel agonists (FPL64176, (S)-Bay K8644, and (R/S)-Bay K8644), cannabinoid receptor agonists (GP1a, and HU308), and uncategorized compounds (antrafenine, buclizine, calpeptin, clomifene, FGIN-1-27, GR46611, guanabenz, lucanthone, luliconazole, pimecrolimus, retapamulin, SR59230A, and tolvaptan).

To identify inhibitors of *T. cruzi* growth for further characterization and development, a high-throughput screening of 2422 pharmacologically annotated bioactive small molecules was conducted against the intracellular amastigote form of *T. cruzi* using high content image-based technology. The calculated Z’ of the assay was 0.548, and a total of 30 compounds were selected using thresholds of >85% parasite growth inhibition and >85% host cell viability, (Fig. 1, Fig. 2). Representative fluorescence images of infected host cells under negative control, positive control, and compound-treated conditions are shown in Fig. 1C, D, and 1E, respectively. All 30 compounds were repurchased and evaluated in dose-response measurements to determine their respective *T. cruzi* activity and selective performance (Supplementary Table S2). Based on their original pharmacological indications, they were classified into TGF-β receptor inhibitors, L-type Ca^2+^ channel agonists, serotonin-dopamine antagonists, cannabinoid receptor agonists, and uncategorized compounds. Among the TGF-β receptor inhibitors, LY2109761 showed the highest activity (IC_50_ = 1.21 ± 0.183 μM) with a selective index (SI) of 41.1. Lurasidone (IC_50_ = 2.17 ± 0.374 μM and SI = 23.0) within the serotonin-dopamine antagonists, and FPL64176 (IC_50_ < 0.0976 μM and SI > 512) among L-type Ca^2+^ channel agonists also showed notable activity with good SI. Pharmacologically uncategorized compounds with activity higher than the reference compound benznidazole included calpeptin (IC_50_ < 0.0976 μM, SI > 258), antrafenine (IC_50_ = 1.20 ± 0.168 μM, SI = 41.4), and luliconazole (IC_50_ < 0.0976 μM and SI > 512) (Supplementary Table S2).

### 3.2 Impact of quantification methods and incubation time on inhibitor potency and kinetics

**Fig. 3:**
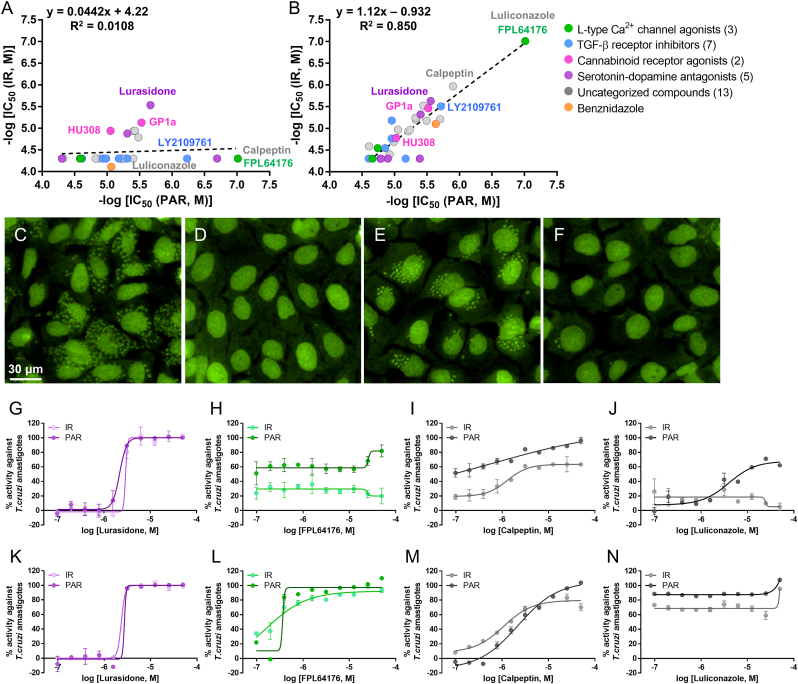
Analysis of 30 hit compounds from intracellular *T. cruzi* Y amastigote assays showing PAR and infection ratio IR after 48 and 120 h of incubation. Correlation plots show IC_50_ values based on IR and PAR at 48 h (A) and 120 h (B). Representative images show infected U2OS cells treated with 0.5% DMSO (C, negative control), 400 μM benznidazole (D, positive control, 72 h), and 0.097 μM luliconazole at 48 h (E) and 120 h (F). Dose-response curves of % IR and PAR activity are shown for lurasidone, FPL64176, calpeptin, and luliconazole after 48 h (G to J) and 120 h (K to N). Scale bar = 30 μm.

**Supplementary Figure 1:**
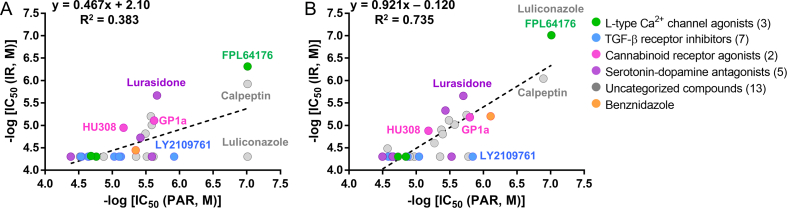
Intracellular *T. cruzi* Y amastigote assays in U2OS cells treated with 30 representative hits for 72 and 96 h. Correlation plots showing IC_50_ values of IR and PAR at 72 h (A) and 96 h (B).

The activities and drug action of the hits identified above were further characterized and compared using different quantification methods. To compare compound efficacy, we assessed both inhibition of parasite proliferation and complete clearance of parasites from infected cells. IC_50_ values were determined using two parameters: the average number of amastigotes per host cell (PAR) and the infection ratio (IR), defined as the proportion of infected versus uninfected host cells (Supplementary Tables S1–4). Representative fluorescence images of DMSO-treated cells (negative control) and benznidazole-treated cells (positive control) are shown in Fig. 3C and D, respectively, from which PAR and IR values are extracted for quantification. To evaluate the kinetics of action, IC_50_ values for PAR and IR were measured after 48, 72, 96, and 120 h of compound incubation. Following 48 h of incubation, the coefficient of determination (R^2^) between the pIC_50_ values derived from IR and PAR was 0.0108, showing essentially no correlation in between both readouts (Fig. 3A). However, with increased exposure times to 72 h, 96 h, and 120 h, the R^2^ values increased to 0.383, 0.735, and 0.850, respectively (Fig. 3B, Supplementary Fig. S1A and B). At 48 h of incubation, only two compounds, lurasidone and GP1a, showed IC_50_ values below 10 μM when IR-based quantification (IC_50_ (IR)) was applied, whereas 28 compounds were active with PAR-based IC_50_ values (IC_50_ (PAR)) (Fig. 3A; Supplementary Table S1). FPL64176, antrafenine, and calpeptin were remarkably active with IC_50_ < 0.0976 μM, respectively, with PAR-based IC_50_ values but were completely inactive even at the highest tested concentration of 50 μM when IR was used to quantify the activity (Fig. 3A and H-I; Supplementary Table S1). While these compounds markedly reduced the total parasite number, they were unable to completely clear the infection from host cells. Lurasidone (IC_50_ (PAR) = 2.15 ± 0.226 μM, IC_50_ (IR) = 2.93 ± 0.281 μM) and GP1a (IC_50_ (PAR) = 2.97 ± 0.419 μM, IC_50_ (IR) = 7.39 ± 1.81 μM at 48 h of incubation) were located near the equipotency line, indicating that the compounds were active regardless of the quantification methods used (Fig. 3A, Supplementary Fig. S1A and B). At 120 h of incubation, all compounds had similar IC_50_ values between calculations based on PAR and IR, as indicated by an R^2^ of 0.850 (Fig. 3B). Compared with inhibition values at different timepoints, some compounds such as lurasidone showed a no or negligible variations in IC_50_ values (2.79 μM with 120 h incubation vs. 2.15 μM with 48 h) (Fig. 3G and K). However, some compounds that showed high reduction in PAR but not in IR at 48 h, such as calpeptin, FPL64176, and LY2109761, were found active in terms of IR reduction at 120 h, implying that with increased incubation time the compounds could clear the parasites from the host (Fig. 3H–I, L-M, Supplementary Fig. S1A and B). Another compound with a dramatic shift in activity depending on incubation time was luliconazole, which showed essentially no effect at 48 h in terms of both IR and PAR, but became increasingly potent with 96 and 120 h of incubations, with the IC_50_ below 97.6 nM (Fig. 3E–F, J, and N).

### 3.3 Comparative susceptibility profiling between *T. cruzi* Y and Dm28c strains

**Fig. 4:**
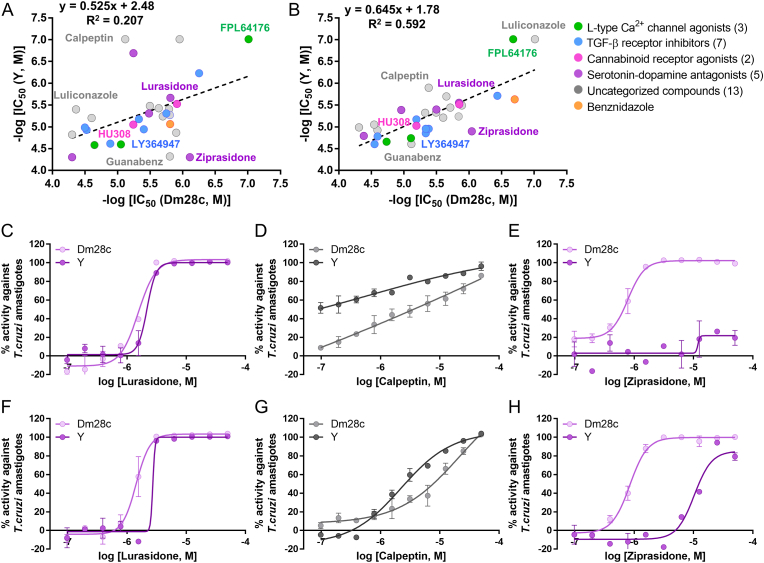
Correlation plots of IC_50_ values based on PAR from intracellular amastigote assays of *T. cruzi* Y and Dm28c strains in U2OS cells treated with 30 representative hits after 48 h (A) and 120 h (B). Dose-response curves based on IR and PAR activity against intracellular amastigotes of *T. cruzi* Y and Dm28c in U2OS cells treated with lurasidone, calpeptin, and ziprasidone at 48 h (C, D, E) and 120 h (F, G, H), respectively.

**Supplementary Figure 3:**
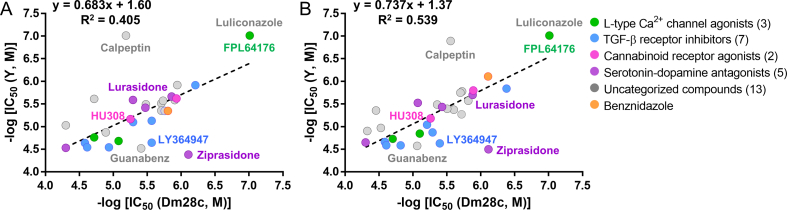
Correlation plots of IC_50_ values of PAR from intracellular amastigotes assays of *T. cruzi* Y and Dm28c strains in U2OS cells treated with 30 representative hits for 72 h (A) and 96 h (B).

Using the same set of compounds, we then investigated the correlation of activity between the two *T. cruzi* strains used in this study, Y and Dm28c. The R^2^ values between the pIC_50_ (PAR) of the Y and Dm28c strains were 0.207 for 48 h and 0.592 for 120 h (Fig. 4A and B). Based on the slope of the linear-regression line (0.405 for 72 h and 0.539 for 96 h), the Dm28c strain was relatively more sensitive than the Y strain to most compounds compared to the Y strain (Fig. 4A–B, Supplementary Fig. S3A and B). Lurasidone showed similar IC_50_ (PAR) values of 2.79 ± 0.315 μM with the Y strain and 1.42 ± 0.104 μM with the Dm28c strain at 120 h of incubation. However, calpeptin was significantly more active against the Y strain (IC_50_ (PAR) = 1.26 ± 0.258 μM) than the Dm28c strain (IC_50_ (PAR) = 4.17 ± 0.492 μM). Conversely, ziprasidone had more potent activity against the Dm28c strain (0.909 ± 0.0933 μM) than the Y strain (12.7 ± 2.73 μM) (Fig. 4C–H; Supplementary Tables S4 and 8).

### 3.4 Stage-specific activity profiling across the *T. cruzi* life cycle

**Fig. 5:**
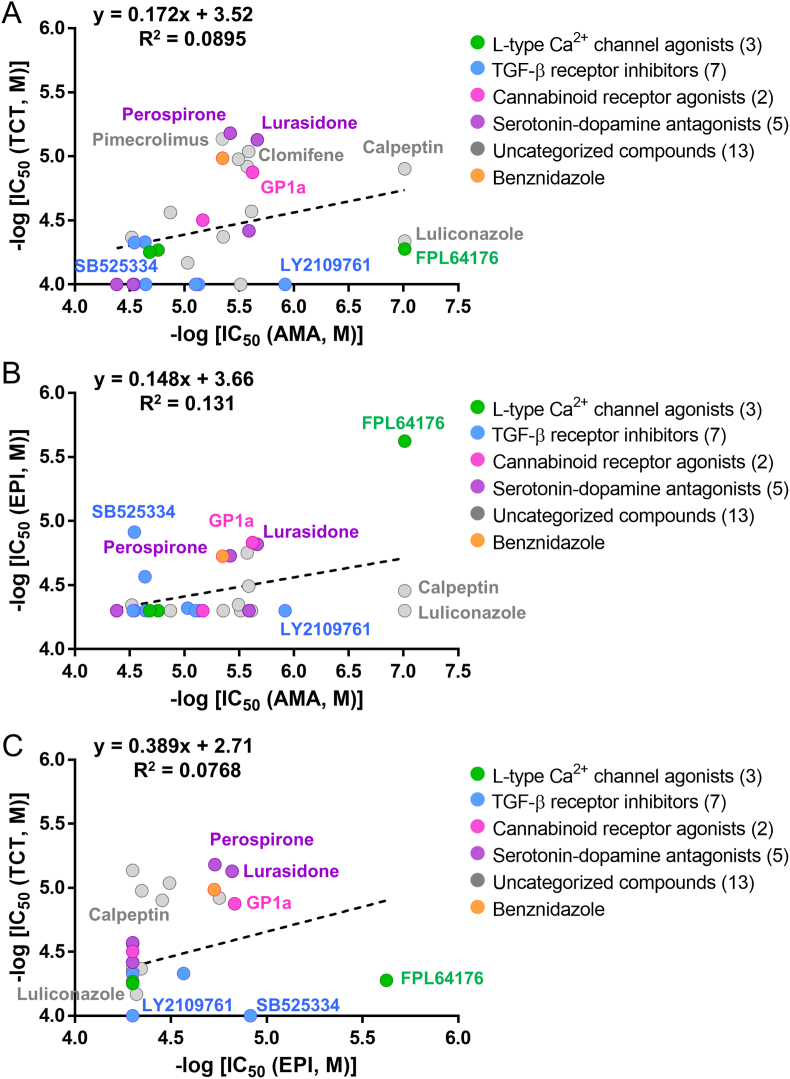
Comparison of IC_50_ values of *T. cruzi* Y for (A) trypomastigotes at 24 h *versus* intracellular amastigote PAR at 72 h, (B) epimastigotes at 72 h *versus* intracellular amastigote PAR at 72 h, and (C) trypomastigotes at 24 h *versus* epimastigotes at 72 h. The data are from treatment with 30 representative selected hits.

We further characterized the activity of compounds in different stages of *T. cruzi* parasite namely epimastigotes and trypomastigotes. A total of 17 compounds showed activity against the trypomastigote form (IC_50_ < 50 μM), with potency generally lower than that observed against the intracellular amastigote form (Fig. 5A; Supplementary Table S9). The two most active compounds were perospirone (IC_50_ = 6.59 ± 0.181 μM) and lurasidone (IC_50_ = 7.43 ± 0.747 μM), whereas other serotonin-dopamine antagonists were inactive (Fig. 5A). Structurally, these two actives are relatively more similar than other compounds within the class, as they share isoindole-1,3(2H)-dione moiety with the same number of carbon linkers (Fig. 2). Pimecrolimus (IC_50_ = 7.32 ± 0.738 μM) displayed slightly higher activity than the benznidazole control (IC_50_ = 10.3 ± 1.35 μM), whereas clomifene (IC_50_ = 9.16 ± 0.394 μM) exhibited comparable potency. In contrast, TGF-β receptor inhibitors and L-type Ca^2+^ channel agonists, which were active against the amastigote form of *T. cruzi* parasite, were not active in trypomastigotes (Fig. 5A; Supplementary Table S9).

A total of 12 compounds showed activity (IC_50_ < 50 μM) against the epimastigote form of the parasite, with only two compounds measured as significantly more potent than benznidazole (IC_50_ = 18.7 ± 1.51 μM) (Fig. 5B; Supplementary Table S9). The most potent compound was FPL64176 (IC_50_ = 2.37 ± 0.126 μM) which was however proven to be significantly more potent against the amastigote form of *T. cruzi* (IC_50_ < 0.0976 μM). SB525334 (IC_50_ = 12.1 ± 0.974 μM) was the only compound to be more potent to suppress the growth of epimastigotes than intracellular amastigotes, whereas all the other TGF-β receptor inhibitors were inactive against the former stage of the parasite (Fig. 5B). Comparing activities in epimastigotes and trypomastigotes, most compounds were more active in trypomastigotes, with SB525334 and FPL64176 being the only ones showing higher activity in epimastigotes (Fig. 5C). Compounds that inhibited all forms were perospirone, lurasidone, clomifene, SR59230A, calpeptin, GP1a, lucanthone, and the control compound benznidazole, while 18 compounds, including most TGF-β receptor inhibitors, were mostly active in the amastigote form (Fig. 5A and B). In general, there was a lack of correlation among the compound potency measurements obtained from the three different assay systems.

### 3.5 Morphological profiling of inhibitor perturbed *T. cruzi* using “Parasite Painting” approach

**Fig. 6:**
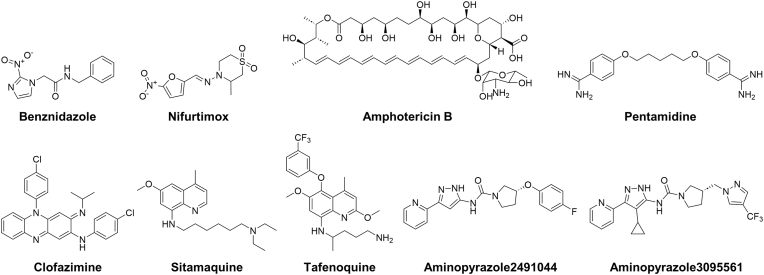
Chemical structures of anti-kinetoplastid reference compounds used for parasite painting alongside the screening hits.

**Fig. 7:**
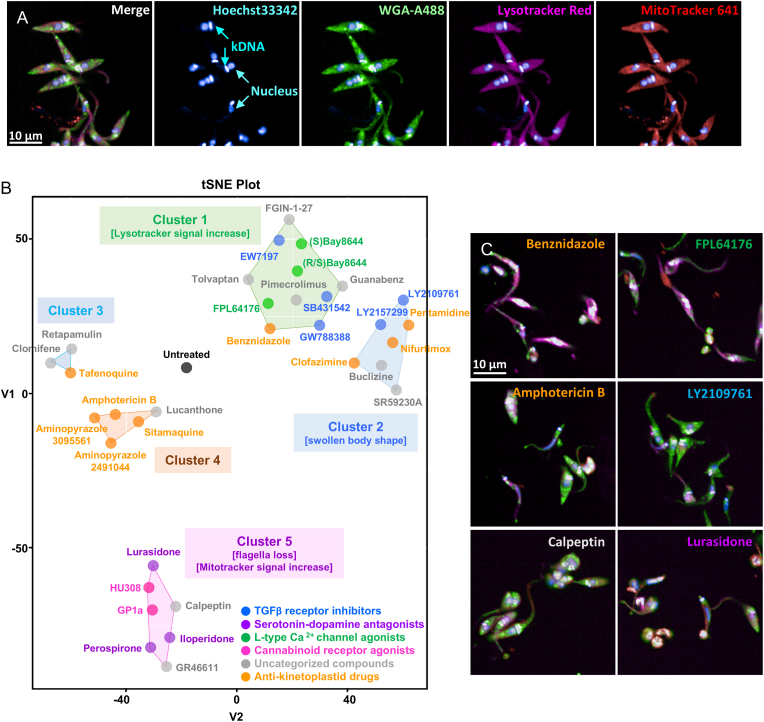
Parasite painting and dimensionality reduction analysis. (A) Images of *T. cruzi* Y trypomastigotes stained with Hoechst 33342, WGA-Alexa488, Lysotracker Red, and Mitotracker Deep Red 641. (B) t-SNE plot from quantified morphological features derived by compound perturbation. (C) Selected images of *T. cruzi* Y trypomastigotes treated with benznidazole, FPL64176, amphotericin B, LY2109761, calpeptin, and lurasidone for 24 h. Scale bar = 10 μm.

**Supplementary Figure 5:**
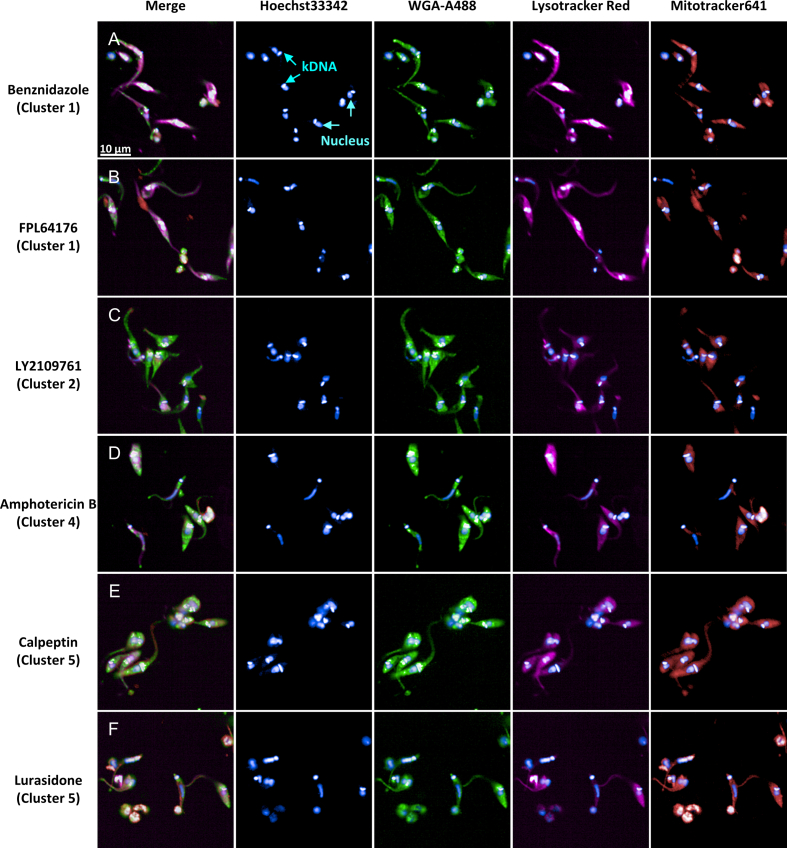
Images of *T. cruzi* Y trypomastigotes stained with Hoechst 33342, WGA-Alexa488, Lysotracker Red, and Mitotracker Deep Red 641 after treatment with (A) 25 μM benznidazole, (B) 25 μM FPL64176, (C) 3.25 μM amphotericin B, (D) 50 μM LY2109761, (E) 3.12 μM calpeptin, and (F) 6.25 μM lurasidone for 24 h. Scale bar = 10 μm.

To analyze the morphological perturbations induced by compounds inhibiting *T. cruzi* parasite growth and classify them according to phenotypically common features, so called cell painting methodology was applied to the trypomastigote form of the parasite. Trypomastigotes were plated as duplicates and treated with compounds at their IC_50_ concentrations for 24 h. Following treatment, mitochondria, lysosomes, nuclei, kinetoplast and parasite surfaces were stained using fluorescently labeled probes. The parasites were then fixed and imaged using four different wavelengths. Reference drugs showing activity against the trypomastigote form of the parasite (IC_50_ ≤ 50 μM) including benznidazole, nifurtimox, pentamidine, clofazimine, amphotericin B, sitamaquine, tafenoquine, and aminopyrazoles were selected and tested together with the investigational compounds identified as inhibitors of the growth of trypomastigote parasite in this analysis (Fig. 6; Supplementary Table S11). The t-distributed stochastic neighbor embedding (t-SNE) based dimensionality reduction of morphological features quantified from the images of fluorescently stained parasites showed five clusters (referred as clusters 1-5) distinct from the untreated control (Fig. 7A). The clinically used drug to treat Chagas disease, benznidazole, clustered with the L-type Ca^2+^ channel agonists FPL64176, (S)-Bay K 8644, and (R/S)-Bay K 8644, as well as with three TGF-β receptor inhibitors EW7197, GW788388, and SB431542 (Fig. 2, Fig. 7B). Parasites in this cluster exhibited increased Lysotracker signal near the nucleus and kinetoplast DNA (Supplementary Fig. S5). In the adjacent cluster 2, another anti-*Trypanosoma* drug, nifurtimox, was grouped with two additional TGF-β receptor inhibitors, LY2109761 and LY5127299. These two compounds share a specific 4-[3-(2-pyridinyl)-1H-pyrazol-4-yl]-quinoline scaffold and are structurally more similar to each other. Parasites in this cluster displayed a swollen body shape without apparent changes in signal from other stains (Fig. 7C, Supplementary Fig. S5). In cluster 4, aminopyrazole compounds were grouped with stimaquine, while another 8-aminoquinoline, tafenoquine, was located in the neighboring cluster 3 (Fig. 7C). In cluster 5, structurally related serotonin-dopamine antagonists of lurasidone, perospirone, and iloperidone were clustered with two cannabinoid receptor agonists, HU308 and GP1a (Fig. 7B). Parasites from this cluster generally exhibited loss of flagella, and those treated with serotonin-dopamine antagonists displayed an increase in mitochondrial signal (Fig. 7C, Supplementary Fig. S5).

## 4 Discussion

Due to the nature of CD as a neglected tropical disease, knowledge around CD biology is limited, and no clinically validated drug target is currently available, hampering the systematic development of small molecules through a rational approach. However, phenotypic screening against the intracellular amastigotes of *T. cruzi* with high content and high throughput capabilities has pushed the frontiers in the discovery process, enabling the development of new active molecules and the introduction of novel targets with modes of action studies utilizing cutting-edge technologies.

The initial image-based high-content screening was successfully applied to DAPI-stained wild-type *T. cruzi* intracellular amastigotes. The image analysis simultaneously differentiated the nuclei of hosts and parasites, enabling the identification of active inhibitors without cytotoxicity (Engel et al., 2010). The assay system with a similar protocol was then utilized to screen mid to large size chemical libraries, leading to identification a large collection of anti-*T. cruzi* inhibitors. Notably, the system was utilized in the GSK *T. cruzi* screening cascade, in which 2310 out of 1.8 million compounds were confirmed active with criteria of pIC_50_ > 5 and SI > 10 generating large collection chemical tools and starting points for further discoveries (Peña et al., 2015). Aside from screenings to identify inhibitors, the *in vitro* assay has evolved to increase the predictability of activity *in vivo* and in humans. Based on the failure of azole compounds in clinical trials, one focus area was to assess compounds' ability to clear intracellular parasites with -cidal activity. Time-to-kill, rate-of-kill, and wash-out assays are representative assays to triage and deprioritize compounds such as posaconazole (or CYP-51 inhibitors). For instance, an elegant experiment designed by MacLean et al. showed that recrudescence of slow-replicating *T. cruzi* Silvio X10/7 was observed 44 days after 16 days of continuous exposure to posaconazole *in vitro* (MacLean et al., 2018). Furthermore, live-imaging rate-of-kill assays with E2-Crimson expressing *T. cruzi* quantified the kinetics of killing, indicating that fexinidazole, a compound in clinical trial, did not clear the parasites as rapidly as the first-line drug benznidazole (Svensen et al., 2021). Also, temporal and wash-out assays using the Malaria Venture Pathogen Box have proven effective in prioritizing inhibitors with irreversible action on *T. cruzi* parasites (Sykes et al., 2022). Similar approaches, but with different conditions such as parasite strains and quantification methods, have proven valid in differentiating *in vitro* activity of CYP51 and other relevant inhibitors to more effective compounds (Sykes et al., 2022). In these aspects, we investigated the inhibitory kinetics of the compounds against intracellular amastigote stage of parasites, using two different strains and four incubation times.

This study identified new inhibitors of *T. cruzi* cellular growth through image-based HTS of 2422 pharmacologically annotated compounds. The respective drug action profiles of these identified hits were further characterized under several experimental conditions looking at a variety of quantification methods, exposure kinetics, as well as parasite forms and strains. A total of 30 compounds were confirmed being active against *T. cruzi* intracellular amastigotes, including L-type Ca^2+^ channel agonists, cannabinoid receptor agonists, TGF-β receptor inhibitors, serotonin-dopamine antagonists, along with 13 uncategorized compounds. The potency of inhibitors at 48 h of exposure varied between the quantification methods of the infection ratio and the parasite number per host cell, but the correlation increased up to R^2^ = 0.850 with 120 h of incubation time. At short incubation times, the azole type of compound luliconazole was completely inactive but became highly potent after 120 h of incubation. This aligns with previous studies on posaconazole and other azoles with slow action as a mode of inhibition and reversibility of activities after wash-out (Moraes et al., 2014; Yang et al., 2017; MacLean et al., 2018; Svensen et al., 2021; Sykes et al., 2022). FPL64176 and calpeptin were determined active at 48 h when the reduction of parasite number was applied for activity measurement but when the infection ratio was considered, IC_50_ was over 50 μM. The recent report by Caio et al. identified FPL64176, an agonist of L-type Ca^2+^ channels, and noted as a pan-strain (Y-H10, Sylvio X10/1, and Cl Brener) active and highly selective anti-*T. cruzi* inhibitor (Kunze and Rampe, 1992; Franco et al., 2019). In both studies, the potency of these compounds was found to be > 10 times more potent than the reference compound benznidazole However, the relatively slow killing kinetics observed here suggest that such characteristics of inhibition should be carefully considered in further studies.

The divergence between PAR and IR at early time points may reflect a distinction between cytostatic and cytocidal activity. PAR, which measures the average number of amastigotes per infected host cell, is sensitive to large reductions in parasite burden but can miss cases where only a small number of parasites remain within the host cell, a situation that is instead captured by IR. Therefore, evaluating IC_50_ values derived from IR, rather than PAR alone, is important to ensure that apparent compound potency is not overestimated due to residual parasites that escape detection. Furthermore, beyond IC_50_ values that reflect potency, the maximum inhibition achievable with IR-based quantification should also be assessed, as it reflects the efficacy of a compound in completely eliminating the parasite from host cells. Compounds that fail to reach full inhibition by IR, regardless of their apparent potency by PAR, should be carefully evaluated, as incomplete clearance of infected host cells leaves a residual parasite population that retains the potential for recrudescence.

In terms of susceptibility difference due to strain difference, the activity correlation was moderate with an R^2^ of 0.592 at 120 h of incubation. Calpeptin was found to be more active in the Y strain, whereas ziprasidone, another serotonin-dopamine antagonist, was more selective towards the Dm28c strain. In fact, ziprasidone was previously identified as a fast-acting-cidal compound in the Sylvio X-10 strain and the potency in the Dm28c strain from this study was similar with a single-digit IC_50_ value in the micromolar range (De Rycker et al., 2016). However, there was a discrepancy in activity with the Y strain and this is possibly due to the fact that Sylvio X-10 and Dm28c belong to the same discrete typing unit I (DTU I), whereas the Y strain belongs to DTU II (Cosentino and Agüero, 2012). Not only ziprasidone but also LY364947 and guanabenz showed over a 5 times difference in IC_50_ values between the strains, reflecting the importance of evaluating compound susceptibility in various DTUs of *T. cruzi* for inhibitor development, especially given that such variations may stem from the intrinsic differences in replication speeds between different DTUs (Neal and van Bueren, 1988; Zingales et al., 2014; Resende et al., 2020; Nicola et al., 2021).

To extend the profiling study of compound activity, the “cell painting” approach was applied to *T. cruzi* parasites for the first time, where morphometric perturbation due to compound exposure is quantified and analyzed to predict potential mechanisms of action of compounds. The method was successful in predicting and searching for compounds with specific mechanisms of action or drug targets, evaluating toxicity, exploring complex disease signatures, modeling diseases, and repurposing drugs (Bray et al., 2016; Christoforow et al., 2019; Foley et al., 2020; Hughes et al., 2020; Laraia et al., 2020; Way et al., 2021; Grigalunas et al., 2021; Liu et al., 2021; Schneidewind et al., 2021; Kelley et al., 2023; Moshkov et al., 2023). *T. cruzi* trypomastigotes treated with various compounds were labeled with four distinct fluorescent dyes and imaged for phenotypic analysis. Dimensionality reduction using t-SNE, followed by clustering, effectively grouped compounds with similar biological classes. For example, all serotonin-dopamine antagonists and cannabinoid receptor agonists were assigned to cluster 5, while two structurally similar aminopyrazoles clustered together with amphotericin B. All L-type Ca^2+^ channel agonists were included in cluster 1, whereas TGF-β receptor inhibitors were distributed across clusters 1 and 2.

Interestingly, the reference compounds benznidazole and nifurtimox, which are structurally similar and share a mode of action involving activation by trypanosomal Type I Nitroreductases, were separated into clusters 1 and 2 (Hall et al., 2011; Hall and Wilkinson, 2012). This observation suggests that compounds within these clusters may induce similar morphological perturbations linked to cell death pathways (Laraia et al., 2020; Dahlin et al., 2023). However, a key limitation of this “painting” approach is the challenge in interpreting phenotypes. In contrast to linear dimensionality reduction methods such as principal component analysis, t-SNE does not readily allow for tracing cluster-specific patterns back to the original features, thereby limiting mechanistic insights into compound- or cluster-specific death phenotypes. Additionally, acquiring high-quality images of *T. cruzi* trypomastigotes posed a technical challenge compared to mammalian cell lines, due to the parasites’ small size and non-adherent nature. The features extracted from these images are generally less sophisticated than those obtained from mammalian cells, as only large morphological changes are typically captured. A further limitation concerns the parasite life stage used in this study. While trypomastigotes are experimentally tractable, the physiologically relevant form in human hosts is the intracellular amastigote (Martín-Escolano et al., 2022). Although labeling amastigotes within adherent host cells is technically feasible, distinguishing parasite-specific features from host cell backgrounds during image analysis presents a critical bottleneck. To address these challenges, future work could employ axenic amastigotes combined with high-resolution microscopy to improve feature resolution without extracting host cell features (Rondinelli et al., 1988; Ferreira et al., 2012). Beyond improvements in image acquisition, advancing the analytical pipeline from population-averaged to single-parasite feature profiles would substantially enhance mechanistic interpretability. This approach is conceptually analogous to single-cell RNA sequencing (scRNA-seq), where genes statistically enriched or depleted in each cluster are identified to characterize cluster-defining biological signatures. In the same way, single-parasite level analysis would enable identification of morphological features that are statistically enriched or depleted between clusters, providing a data-driven description of the phenotypic signatures that define each cluster and generating testable hypotheses for follow-up mechanistic studies. We are currently developing this single-parasite level pipeline using other protozoan parasites where imaging is technically less challenging. Despite these limitations, this study represents the first application of the “painting” method to protozoan parasites. Refining the image acquisition strategy and expanding the reference library to include bioactive compounds with well-characterized anti-*T. cruzi* mechanisms would further enhance predictive accuracy for determining modes of action of novel compounds.

One of the interesting classes of compounds profiled from this study was the serotonin-dopamine antagonists. They are antipsychotic medications used to treat schizophrenia and bipolar disorder. Within the class, five compounds sharing either a benzisothiazole or benzisoxazole were found active (Fig. 2). Among them, lurasidone and perospirone, which share a cyclohexanedicarboximide on the other side of the benzisothiazole, were found active in all forms of *T. cruzi* (amastigote, trypomastigote, and epimastigote). Especially, lurasidone was able to completely eliminate amastigotes with a short exposure time of 48 h. A previous study screening FDA-approved drugs identified the psychotropic drugs fluoxetine, paroxetine, and sertraline, but they are all structurally distinct from those in this study (Planer et al., 2014). Since there are no serotonin or dopamine receptors present in *T. cruzi*, pharmacophores from lurasidone and perospirone may serve as unique templates for new molecular entities with optimized activity in *T. cruzi*.

In terms of translational prospects, our *in vitro* profiling pipeline was specifically designed to address key pitfalls that led to clinical failures of azole-class compounds in Chagas disease. Extended incubation assays (48–120 h) comparing PAR and IR readouts revealed that luliconazole, like the clinical-failure compound posaconazole, displays slow-acting kinetics and achieves parasite clearance only after prolonged exposure, a phenotype that historically predicts poor *in vivo* efficacy. Conversely, lurasidone exhibited rapid and complete parasite clearance within 48 h by both PAR and IR metrics, with consistent multi-stage activity across amastigotes, trypomastigotes, and epimastigotes, and a satisfactory SI of 23.0. As an approved therapeutic with well-characterized pharmacokinetics, lurasidone represents a compelling candidate for further evaluation. In the parasite painting dimensionality reduction space, lurasidone was placed in Cluster 5, clearly distinct from benznidazole, indicating a different and potentially novel mechanism of action that may be worth pursuing as a proof-of-concept *in vivo* study. FPL64176, while exceptionally potent and highly selective (IC_50_ < 0.097 μM, SI > 512), presents pharmacological concerns as a Ca^2+^ channel agonist that warrant mechanistic deconvolution prior to *in vivo* progression. In the same dimensionality reduction space, FPL64176 clustered together with benznidazole, an approved first-line drug for Chagas disease, suggesting that both compounds may induce overlapping morphological perturbations and potentially share downstream cellular consequences, which adds phenotypic support to its further investigation.

In conclusion, several new anti-*T. cruzi* chemotypes were identified through HTS, some of which are already approved for clinical use in other indications. Extensive profiling of the activity in terms of kinetics of action, and different parasite forms and strains have further deciphered the characteristics of drug action with regard to a variety of parameters relevant to *T. cruzi* parasite. Additionally, the “cell painting” methodology was first applied to protozoan parasites, and its potential for use in classifying inhibitors based on morphological parameters was examined. Taken together, the series of assays introduced in this study allows for an advanced understanding of inhibitors for further development against CD.

## CRediT authorship contribution statement

**Kyung-Hwa Baek:** Writing – original draft, Project administration, Methodology, Investigation, Formal analysis, Data curation, Conceptualization. **Hyeryon Lee:** Methodology, Formal analysis. **Sooyoung Byun:** Methodology, Formal analysis. **Olga Genilloud:** Writing – review & editing, Funding acquisition, Conceptualization. **Jean-Robert Ioset:** Writing – review & editing, Supervision, Funding acquisition, Conceptualization. **David Shum:** Writing – review & editing, Funding acquisition, Conceptualization. **Joo Hwan No:** Writing – review & editing, Writing – original draft, Supervision, Project administration, Investigation, Funding acquisition, Conceptualization.

## Ethical statement

Not required.

## Funding

This work was supported by the National Research foundation of Korea (NRF) grant funded by the Korea government (MSIT program) (RS-2024-00398073 and RS-2025-00563140) and by “la Caixa” Foundation grant number HR20-00584.

## Declaration of competing interest

The authors declare no conflicts of interest.

## Figures and Tables

**Supplementary Figure 2:**
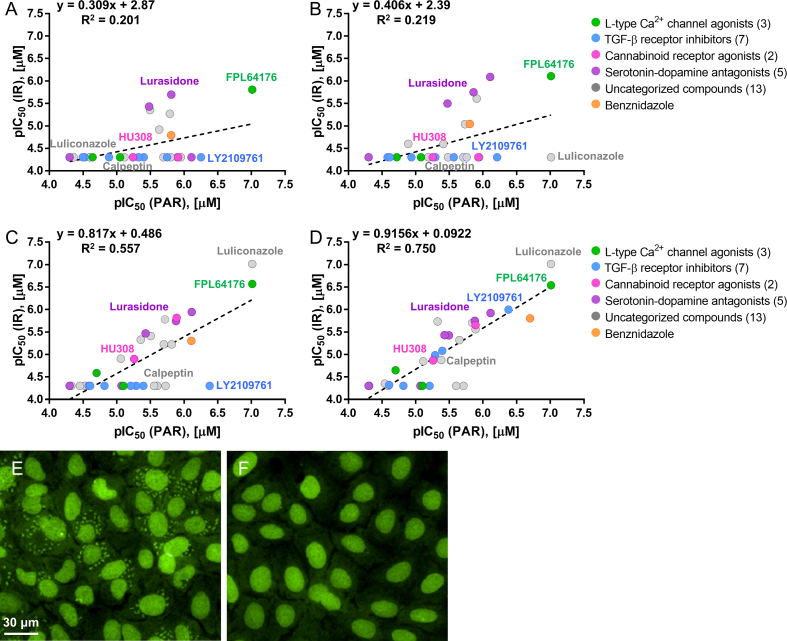
Intracellular *T. cruzi* Dm28c amastigote assays in U2OS cells treated with 30 representative hits for 48 to 120 h. Correlation plots showing IC_50_ values of IR and PAR at 48 (A), 72 (B), 96 (C), and 120 h (D). Images of infected U2OS cells treated with 0.5% DMSO (E, negative control) and 400 μM benznidazole (F, positive control) for 72 h. Scale bar = 30 μm.

**Supplementary Figure 4:**
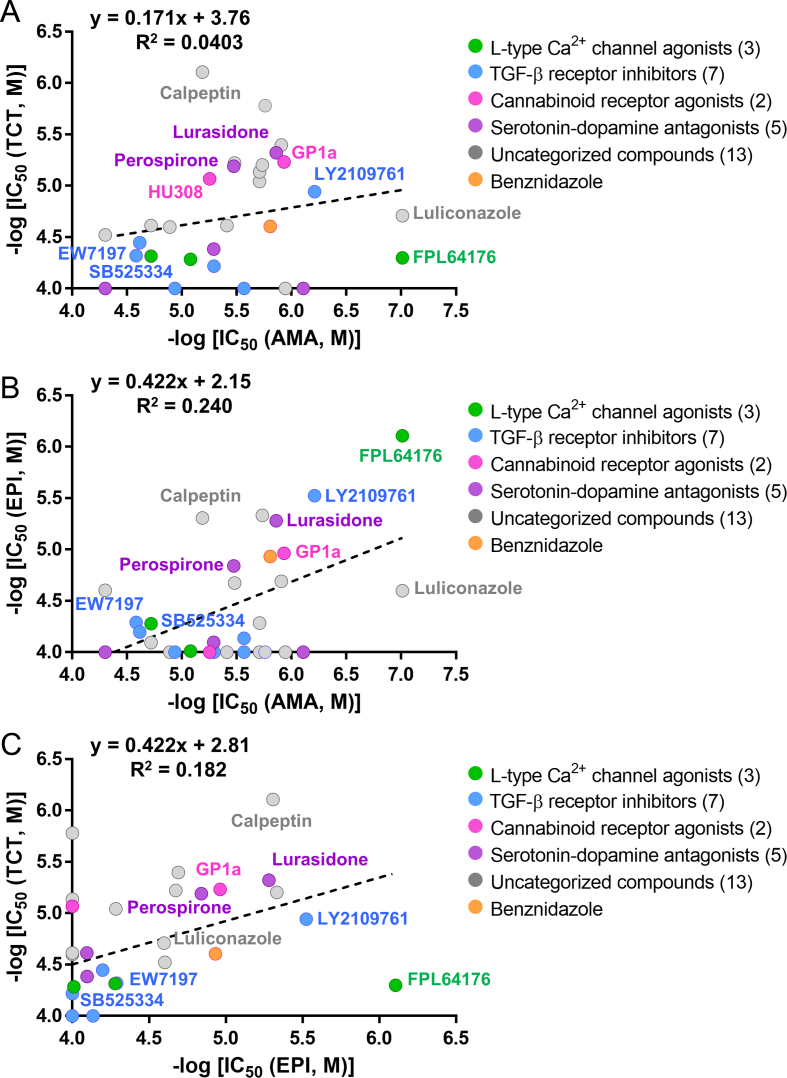
Comparison of IC_50_ values of *T. cruzi* Dm28c for (A) trypomastigotes at 24 h versus PAR of intracellular amastigotes at 72 h, (B) epimastigotes at 72 h versus PAR of intracellular amastigotes at 72 h, and (C) trypomastigotes at 24 h versus epimastigotes at 72 h following treatment with 30 selected hits.
