# Supplementary material for: In vitro profiling of Trypanosoma cruzi inhibitors identified from High throughput Screening and application to parasite painting
Source: Int J Parasitol Drugs Drug Resist. 2026 May 14;31:100651. doi: 10.1016/j.ijpddr.2026.100651 (PMC13213233; doi:10.1016/j.ijpddr.2026.100651)
Supplement: Multimedia component 1 [file mmc1.docx]

**Supplementary Table S1.** IC_50_ values of PAR, IR, CC_50_ values in U2OS cells, and selectivity indices (SI) for 30 selected hits tested in *T. cruzi* Y intracellular amastigote assays after 48 hours treatment.

| **No.** | **Compound** | **PAR (μM)** | **IR (μM)** | **CC_50_ (μM)** | **SI (CC_50_/PAR)** |
| --- | --- | --- | --- | --- | --- |
| 1 | LY2109761 | 0.589 ± 0.397 | > 50 | > 50 | 84.9 |
| 2 | LY2157299 | 4.90 ± 3.30 | > 50 | > 50 | 10.1 |
| 3 | LY364947 | 11.4 ± 9.86 | > 50 | > 50 | 4.39 |
| 4 | GW788388 | 6.65 ± 0.712 | > 50 | > 50 | 7.52 |
| 5 | EW7197 | 24.3 ± 3.61 | > 50 | 25.8 ± 1.65 | 1.06 |
| 6 | SB431542 | 11.7 ± 1.04 | > 50 | > 50 | 4.25 |
| 7 | SB525334 | 10.3 ± 1.30 | > 50 | > 50 | 4.81 |
| 8 | Lurasidone | 2.15 ± 0.226 | 2.93 ± 0.281 | > 50 | 23.1 |
| 9 | Perospirone | 4.90 ± 0.546 | 13.1 ± 2.72 | 13.4 ± 1.55 | 2.75 |
| 10 | Ziprasidone | > 50 | > 50 | > 50 | 1 |
| 11 | Iloperidone | 0.203 ± 0.210 | > 50 | > 50 | 246 |
| 12 | Risperidone | > 50 | > 50 | > 50 | 1 |
| 13 | FPL64176 | < 0.0976 | > 50 | > 50 | > 512 |
| 14 | (S)-Bay K8644 | 26.2 ± 2.00 | > 50 | > 50 | 1.91 |
| 15 | (R/S)-Bay K8644 | 25.3 ± 5.82 | > 50 | > 50 | 1.97 |
| 16 | GP1a | 2.97 ± 0.419 | 7.39 ± 1.81 | 6.25 ± 0.308 | 2.10 |
| 17 | HU308 | 8.91 ± 3.43 | 11.4 ± 0.981 | 13.6 ± 3.74 | 1.53 |
| 18 | Antrafenine | < 0.0976 | > 50 | > 50 | > 512 |
| 19 | Buclizine | 5.72 ± 1.70 | > 50 | > 50 | 8.73 |
| 20 | Calpeptin | < 0.0976 | > 50 | 25.0 ± 1.68 | 256 |
| 21 | Clomifene | 3.93 ± 0.741 | 11.5 ± 2.32 | 6.73 ± 0.741 | 1.71 |
| 22 | FGIN-1-27 | 13.6 ± 2.25 | > 50 | > 50 | 3.67 |
| 23 | GR46611 | 6.25 ± 1.09 | > 50 | 26.4 ± 2.76 | 4.24 |
| 24 | Guanabenz | 47.8 ± 7.48 | > 50 | > 50 | 1.04 |
| 25 | Lucanthone | 3.74 ± 0.621 | 11.4 ± 4.25 | 3.43 ± 0.442 | 0.92 |
| 26 | Luliconazole | 3.97 ± 1.12 | > 50 | > 50 | 12.5 |
| 27 | Pimecrolimus | 5.37 ± 0.491 | > 50 | 5.34 ± 1.38 | 1 |
| 28 | Retapamulin | > 50 | > 50 | > 50 | 1 |
| 29 | SR59230A | 3.31 ± 0.618 | 16.2 ± 1.23 | 10.9 ± 4.59 | 3.29 |
| 30 | Tolvaptan | 15.0 ± 2.08 | > 50 | > 50 | 3.32 |
| Ref | Benznidazole | 8.65 ± 4.51 | 66.8 ± 7.52 | > 400 | 46.2 |

**Supplementary Table S2.** IC_50_ values of PAR, IR, CC_50_ values in U2OS cells, and SI for 30 selected hits tested in *T. cruzi* Y intracellular amastigote assays after 72 hours treatment.

| **No.** | **Compound** | **PAR (μM)** | **IR (μM)** | **CC_50_ (μM)** | **SI (CC_50_/PAR)** |
| --- | --- | --- | --- | --- | --- |
| 1 | LY2109761 | 1.21 ± 0.183 | > 50 | > 50 | 41.1 |
| 2 | LY2157299 | 7.48 ± 0.780 | > 50 | > 50 | 6.68 |
| 3 | LY364947 | 22.6 ± 3.04 | > 50 | > 50 | 2.20 |
| 4 | GW788388 | 7.96 ± 1.20 | > 50 | > 50 | 6.28 |
| 5 | EW7197 | 23.0 ± 3.05 | > 50 | 24.9 ± 8.50 | 1.08 |
| 6 | SB431542 | 28.7 ± 2.56 | > 50 | > 50 | 1.74 |
| 7 | SB525334 | 28.7 ± 2.17 | > 50 | > 50 | 1.74 |
| 8 | Lurasidone | 2.17 ± 0.374 | 2.16 ± 0.347 | > 50 | 23.0 |
| 9 | Perospirone | 3.82 ± 0.405 | 18.8 ± 4.10 | 13.7 ± 4.70 | 3.60 |
| 10 | Ziprasidone | 41.5 ± 8.39 | > 50 | > 50 | 1.20 |
| 11 | Iloperidone | 2.58 ± 0.418 | > 50 | > 50 | 19.3 |
| 12 | Risperidone | 29.5 ± 3.65 | > 50 | 48.6 ± 4.19 | 1.64 |
| 13 | FPL64176 | < 0.0976 | 0.484 ± 0.292 | > 50 | > 512 |
| 14 | (S)-Bay K8644 | 17.3 ± 3.78 | > 50 | > 50 | 2.88 |
| 15 | (R/S)-Bay K8644 | 20.8 ± 2.04 | 48.0 ± 8.73 | 35.3 ± 2.76 | 1.69 |
| 16 | GP1a | 2.39 ± 0.189 | 7.83 ± 1.74 | 6.12 ± 0.780 | 2.55 |
| 17 | HU308 | 6.82 ± 1.14 | 11.2 ± 1.51 | 9.26 ± 2.05 | 1.36 |
| 18 | Antrafenine | 1.20 ± 0.168 | > 50 | > 50 | 41.4 |
| 19 | Buclizine | 3.08 ± 452 | > 50 | 23.7 ± 6.75 | 7.69 |
| 20 | Calpeptin | < 0.0976 | 1.19 ± 0.691 | 25.1 ± 2.25 | 258 |
| 21 | Clomifene | 2.59 ± 0.379 | 9.81 ± 0.672 | 6.29 ± 0.635 | 2.43 |
| 22 | FGIN-1-27 | 4.44 ± 0.787 | > 50 | > 50 | 11.2 |
| 23 | GR46611 | 13.4 ± 1.48 | > 50 | 32.3 ± 3.42 | 2.40 |
| 24 | Guanabenz | 30.2 ± 4.47 | > 50 | > 50 | 1.65 |
| 25 | Lucanthone | 2.67 ± 0.336 | 6.32 ± 1.09 | 3.05 ± 0.144 | 1.14 |
| 26 | Luliconazole | < 0.0976 | > 50 | > 50 | > 512 |
| 27 | Pimecrolimus | 4.51 ± 0.452 | > 50 | 6.79 ± 0.652 | 1.50 |
| 28 | Retapamulin | 2.45 ± 0.319 | > 50 | > 50 | 20.3 |
| 29 | SR59230A | 3.22 ± 0.516 | 15.2 ± 3.36 | 11.8 ± 1.62 | 3.66 |
| 30 | Tolvaptan | 9.37 ± 1.18 | > 50 | 38.4 ± 1.77 | 4.10 |
| Ref | Benznidazole | 4.49 ± 0.862 | 35.9 ± 3.69 | > 400 | 88.9 |

**Supplementary Table S3.** IC_50_ values of PAR, IR, CC_50_ values in U2OS cells, and SI for 30 selected hits tested in *T. cruzi* Y intracellular amastigote assays after 96 hours treatment.

| **No.** | **Compound** | **PAR (μM)** | **IR (μM)** | **CC_50_ (μM)** | **SI (CC_50_/PAR)** |
| --- | --- | --- | --- | --- | --- |
| 1 | LY2109761 | 1.44 ± 0.132 | > 50 | > 50 | 34.5 |
| 2 | LY2157299 | 13.4 ± 0.973 | > 50 | > 50 | 3.72 |
| 3 | LY364947 | 23.5 ± 2.79 | > 50 | > 50 | 2.12 |
| 4 | GW788388 | 9.09 ± 0.902 | > 50 | > 50 | 5.50 |
| 5 | EW7197 | 21.8 ± 3.03 | > 50 | 19.9 ± 3.40 | 0.91 |
| 6 | SB431542 | 26.0 ± 0.781 | > 50 | > 50 | 1.92 |
| 7 | SB525334 | 26.0 ± 1.18 | > 50 | > 50 | 1.92 |
| 8 | Lurasidone | 1.99 ± 0.278 | 2.20 ± 0.338 | > 50 | 25.0 |
| 9 | Perospirone | 3.68 ± 0.214 | 4.66 ± 0.591 | 12.8 ± 1.28 | 3.48 |
| 10 | Ziprasidone | 31.8 ± 6.59 | > 50 | > 50 | 1.57 |
| 11 | Iloperidone | 2.98 ± 0.495 | > 50 | > 50 | 16.7 |
| 12 | Risperidone | 22.4 ± 1.69 | > 50 | > 50 | 2.22 |
| 13 | FPL64176 | < 0.0976 | < 0.0976 | > 50 | > 512 |
| 14 | (S)-Bay K8644 | 18.7 ± 1.57 | > 50 | > 50 | 2.67 |
| 15 | (R/S)-Bay K8644 | 14.2 ± 1.06 | > 50 | 25.7 ± 2.45 | 1.80 |
| 16 | GP1a | 1.59 ± 0.126 | 6.62 ± 1.11 | 5.82 ± 0.597 | 3.66 |
| 17 | HU308 | 6.59 ± 0.948 | 13.1 ± 1.46 | 12.8 ± 2.38 | 1.95 |
| 18 | Antrafenine | 1.67 ± 0.112 | > 50 | > 50 | 29.9 |
| 19 | Buclizine | 4.23 ± 0.449 | 12.5 ± 4.24 | 17.1 ± 4.84 | 4.04 |
| 20 | Calpeptin | 0.128 ± 0.0671 | 0.906 ± 0.299 | 25.8 ± 1.53 | 201 |
| 21 | Clomifene | 2.67 ± 0.264 | 9.63 ± 0.519 | 5.83 ± 0.326 | 2.18 |
| 22 | FGIN-1-27 | 4.04 ± 0.518 | 15.7 ± 1.60 | > 50 | 12.3 |
| 23 | GR46611 | 10.6 ± 1.72 | > 50 | 30.9 ± 3.76 | 2.90 |
| 24 | Guanabenz | 26.7 ± 2.03 | 32.9 ± 1.86 | > 50 | 1.87 |
| 25 | Lucanthone | 1.78 ± 0.371 | 5.86 ± 0.375 | 2.73 ± 0.127 | 1.53 |
| 26 | Luliconazole | < 0.0976 | < 0.0976 | > 50 | > 512 |
| 27 | Pimecrolimus | 5.35 ± 0.627 | 25.0 ± 3.51 | 9.18 ± 0.613 | 1.71 |
| 28 | Retapamulin | 4.42 ± 1.28 | > 50 | > 50 | 11.3 |
| 29 | SR59230A | 3.26 ± 0.411 | 7.72 ± 3.13 | 11.6 ± 1.88 | 3.56 |
| 30 | Tolvaptan | 12.5 ± 1.73 | > 50 | 27.8 ± 1.56 | 2.23 |
| Ref | Benznidazole | < 0.782 | 6.25 ± 1.09 | > 400 | > 511 |

**Supplementary Table S4.** IC_50_ values of PAR, IR, CC_50_ values in U2OS cells, and SI for 30 selected hits tested in *T. cruzi* Y intracellular amastigote assays after 120 hours treatment.

| **No.** | **Compound** | **PAR (μM)** | **IR (μM)** | **CC_50_ (μM)** | **SI (CC_50_/PAR)** |
| --- | --- | --- | --- | --- | --- |
| 1 | LY2109761 | 1.93 ± 0.167 | 3.11 ± 0.371 | > 50 | 25.8 |
| 2 | LY2157299 | 13.9 ± 2.05 | 28.6 ± 1.53 | > 50 | 3.57 |
| 3 | LY364947 | 11.1 ± 1.17 | 17.1 ± 0.891 | > 50 | 4.50 |
| 4 | GW788388 | 11.0 ± 1.13 | 6.60 ± 0.194 | > 50 | 4.52 |
| 5 | EW7197 | 16.5 ± 2.49 | > 50 | 14.9 ± 3.16 | 0.90 |
| 6 | SB431542 | 25.0 ± 1.76 | > 50 | > 50 | 2.00 |
| 7 | SB525334 | 6.76 ± 2.28 | > 50 | > 50 | 7.40 |
| 8 | Lurasidone | 2.79 ± 0.315 | 2.35 ± 0.145 | > 50 | 17.9 |
| 9 | Perospirone | 3.98 ± 0.548 | 4.79 ± 0.319 | 13.0 ± 2.56 | 3.29 |
| 10 | Ziprasidone | 12.7 ± 2.73 | > 50 | > 50 | 3.94 |
| 11 | Iloperidone | 4.09 ± 0.782 | > 50 | 27.6 ± 2.79 | 6.76 |
| 12 | Risperidone | 16.1 ± 2.33 | > 50 | > 50 | 3.09 |
| 13 | FPL64176 | < 0.0976 | < 0.0976 | > 50 | > 512 |
| 14 | (S)-Bay K8644 | 21.9 ± 2.85 | > 50 | > 50 | 2.28 |
| 15 | (R/S)-Bay K8644 | 18.1 ± 1.89 | 28.5 ± 1.20 | 26.6 ± 2.55 | 1.47 |
| 16 | GP1a | 3.04 ± 0.179 | 3.45 ± 0.400 | 6.19 ± 2.11 | 2.04 |
| 17 | HU308 | 9.46 ± 1.26 | 16.6 ± 3.21 | 12.0 ± 2.68 | 1.28 |
| 18 | Antrafenine | 3.62 ± 0.322 | 3.02 ± 0.292 | > 50 | 13.8 |
| 19 | Buclizine | 6.13 ± 0.481 | 11.7 ± 0.832 | 11.8 ± 3.27 | 1.93 |
| 20 | Calpeptin | 1.26 ± 0.258 | 1.08 ± 0.179 | 28.5 ± 4.30 | 22.4 |
| 21 | Clomifene | 3.22 ± 0.0869 | 6.71 ± 0.543 | 5.74 ± 0.839 | 1.78 |
| 22 | FGIN-1-27 | 4.69 ± 0.485 | 6.29 ± 0.874 | > 50 | 10.6 |
| 23 | GR46611 | 11.9 ± 2.08 | 40.0 ± 10.0 | 24.2 ± 5.34 | 2.03 |
| 24 | Guanabenz | 24.7 ± 3.19 | 26.1 ± 1.56 | > 50 | 2.02 |
| 25 | Lucanthone | 1.99 ± 0.164 | 6.16 ± 0.789 | 2.66 ± 0.136 | 1.33 |
| 26 | Luliconazole | < 0.0976 | < 0.0976 | > 50 | > 512 |
| 27 | Pimecrolimus | 5.79 ± 0.610 | 10.6 ± 0.459 | 12.0 ± 2.02 | 2.08 |
| 28 | Retapamulin | 8.89 ± 1.26 | 10.6 ± 0.788 | > 50 | 5.62 |
| 29 | SR59230A | 4.76 ± 0.793 | 5.75 ± 0.965 | 11.5 ± 3.12 | 2.43 |
| 30 | Tolvaptan | 10.3 ± 1.54 | 20.7 ± 4.43 | > 50 | 4.84 |
| Ref | Benznidazole | 2.33 ± 0.353 | 7.92 ± 1.14 | > 400 | 171 |

**Supplementary Table S5.** IC_50_ values of PAR, IR, CC_50_ values in U2OS cells, and SI for 30 selected hits tested in *T. cruzi* Dm28c intracellular amastigote assays after 48 hours treatment.

| **No.** | **Compound** | **PAR (μM)** | **IR (μM)** | **CC_50_ (μM)** | **SI (CC_50_/PAR)** |
| --- | --- | --- | --- | --- | --- |
| 1 | LY2109761 | 0.561 ± 0.503 | > 50 | > 50 | 89.1 |
| 2 | LY2157299 | 1.79 ± 0.697 | > 50 | > 50 | 27.9 |
| 3 | LY364947 | 3.97 ± 0.877 | > 50 | > 50 | 12.5 |
| 4 | GW788388 | 4.74 ± 0.830 | > 50 | > 50 | 10.5 |
| 5 | EW7197 | 12.9 ± 0.979 | > 50 | 28.5 ± 9.72 | 2.20 |
| 6 | SB431542 | 30.0 ± 3.44 | > 50 | 47.9 ± 2.18 | 1.60 |
| 7 | SB525334 | 31.7 ± 4.54 | > 50 | > 50 | 1.57 |
| 8 | Lurasidone | 1.54 ± 0.118 | 2.01 ± 0.429 | > 50 | 32.3 |
| 9 | Perospirone | 3.29 ± 0.461 | 3.70 ± 0.587 | 23.3 ± 1.05 | 7.08 |
| 10 | Ziprasidone | 0.779 ± 0.0588 | > 50 | 3.26 ± 0.190 | 4.19 |
| 11 | Iloperidone | 5.74 ± 3.05 | > 50 | > 50 | 8.71 |
| 12 | Risperidone | > 50 | > 50 | > 50 | 1.00 |
| 13 | FPL64176 | < 0.0976 | 1.55 ± 0.229 | 48.0 ± 3.07 | 493 |
| 14 | (S)-Bay K8644 | 22.8 ± 2.55 | > 50 | > 50 | 2.19 |
| 15 | (R/S)-Bay K8644 | 8.95 ± 0.986 | > 50 | 22.0 ± 2.11 | 2.47 |
| 16 | GP1a | 1.23 ± 0.153 | > 50 | 5.71 ± 0.338 | 4.65 |
| 17 | HU308 | 5.75 ± 1.19 | > 50 | 11.9 ± 0.418 | 2.07 |
| 18 | Antrafenine | 1.11 ± 0.169 | > 50 | > 50 | 44.7 |
| 19 | Buclizine | 2.03 ± 0.268 | > 50 | 17.4 ± 2.67 | 8.59 |
| 20 | Calpeptin | 7.65 ± 1.08 | > 50 | > 50 | 6.53 |
| 21 | Clomifene | 1.63 ± 0.153 | 5.34 ± 1.88 | 6.28 ± 0.336 | 3.85 |
| 22 | FGIN-1-27 | 1.26 ± 1.38 | > 50 | > 50 | 39.5 |
| 23 | GR46611 | 25.1 ± 2.72 | > 50 | > 50 | 1.99 |
| 24 | Guanabenz | 1.58 ± 0.478 | > 50 | > 50 | 31.6 |
| 25 | Lucanthone | 2.33 ± 0.291 | 12.1 ± 0.629 | 3.20 ± 0.547 | 1.37 |
| 26 | Luliconazole | 43.5 ± 2.44 | > 50 | 45.0 ± 2.17 | 1.04 |
| 27 | Pimecrolimus | 1.57 ± 0.110 | > 50 | 6.25 ± 1.64 | 3.96 |
| 28 | Retapamulin | > 50 | > 50 | > 50 | 1.00 |
| 29 | SR59230A | 3.21 ± 0.497 | 4.44 ± 0.652 | 14.1 ± 0.948 | 4.40 |
| 30 | Tolvaptan | > 50 | > 50 | > 50 | 1.00 |
| Ref | Benznidazole | 1.56 ± 0.0530 | 16.1 ± 3.47 | > 400 | 255 |

**Supplementary Table S6.** IC_50_ values of PAR, IR, CC_50_ values in U2OS cells, and SI for 30 selected hits tested in *T. cruzi* Dm28c intracellular amastigote assays after 72 hours treatment.

| **No.** | **Compound** | **PAR (μM)** | **IR (μM)** | **CC_50_ (μM)** | **SI (CC_50_/PAR)** |
| --- | --- | --- | --- | --- | --- |
| 1 | LY2109761 | 0.616 ± 0.170 | > 50 | > 50 | 81.1 |
| 2 | LY2157299 | 2.72 ± 1.62 | > 50 | > 50 | 18.3 |
| 3 | LY364947 | 2.71 ± 0.811 | > 50 | > 50 | 18.4 |
| 4 | GW788388 | 5.10 ± 2.17 | > 50 | > 50 | 9.79 |
| 5 | EW7197 | 26.1 ± 2.88 | > 50 | 24.4 ± 8.87 | 0.94 |
| 6 | SB431542 | 24.2 ± 3.49 | > 50 | 48.0 ± 3.46 | 1.99 |
| 7 | SB525334 | 11.5 ± 2.90 | > 50 | > 50 | 4.32 |
| 8 | Lurasidone | 1.37 ± 0.124 | 1.79 ± 0.232 | > 50 | 36.3 |
| 9 | Perospirone | 3.37 ± 0.242 | 3.17 ± 0.419 | 14.9 ± 0.695 | 4.45 |
| 10 | Ziprasidone | 0.781 ± 0.0642 | 0.812 ± 0.149 | 3.36 ± 0.343 | 4.31 |
| 11 | Iloperidone | 5.12 ± 2.23 | > 50 | > 50 | 9.75 |
| 12 | Risperidone | > 50 | > 50 | > 50 | 1.00 |
| 13 | FPL64176 | < 0.0976 | 0.781 ± 0.241 | 47.6 ± 2.98 | 488 |
| 14 | (S)-Bay K8644 | 19.0 ± 3.56 | > 50 | > 50 | 2.62 |
| 15 | (R/S)-Bay K8644 | 8.33 ± 1.17 | > 50 | 19.6 ± 2.80 | 2.36 |
| 16 | GP1a | 1.17 ± 0.122 | > 50 | 5.43 ± 0.402 | 4.62 |
| 17 | HU308 | 5.58 ± 0.935 | > 50 | 10.5 ± 0.510 | 1.90 |
| 18 | Antrafenine | 1.14 ± 0.203 | > 50 | > 50 | 43.7 |
| 19 | Buclizine | 1.95 ± 0.265 | > 50 | 17.0 ± 3.56 | 8.71 |
| 20 | Calpeptin | 6.48 ± 1.98 | > 50 | 47.7 ± 3.01 | 7.36 |
| 21 | Clomifene | 1.23 ± 0.105 | 2.47± 0.726 | 4.46 ± 0.201 | 3.61 |
| 22 | FGIN-1-27 | 1.96 ± 0.620 | > 50 | 47.9 ± 4.64 | 24.4 |
| 23 | GR46611 | 12.8 ± 2.47 | 25.2 ± 2.45 | > 50 | 3.89 |
| 24 | Guanabenz | 3.88 ± 0.611 | 25.2 ± 3.20 | 28.1 ± 1.21 | 7.26 |
| 25 | Lucanthone | 1.84 ± 0.124 | 9.20 ± 0.678 | 2.40 ± 0.117 | 1.30 |
| 26 | Luliconazole | < 0.0976 | > 50 | 49.4 ± 3.04 | 506 |
| 27 | Pimecrolimus | 1.74 ± 0.117 | > 50 | 2.72 ± 0.305 | 1.57 |
| 28 | Retapamulin | 19.0 ± 3.32 | > 50 | > 50 | 2.62 |
| 29 | SR59230A | 3.29 ± 0.238 | > 50 | 12.6 ± 0.680 | 3.84 |
| 30 | Tolvaptan | > 50 | > 50 | > 50 | 1.00 |
| Ref | Benznidazole | 1.56 ± 0.391 | 9.05 ± 1.80 | > 400 | 255 |

**Supplementary Table S7.** IC_50_ values of PAR, IR, CC_50_ values in U2OS cells, and SI for 30 selected hits tested in *T. cruzi* Dm28c intracellular amastigote assays after 96 hours treatment.

| **No.** | **Compound** | **PAR (μM)** | **IR (μM)** | **CC_50_ (μM)** | **SI (CC_50_/PAR)** |
| --- | --- | --- | --- | --- | --- |
| 1 | LY2109761 | 0.416 ± 0.0621 | > 50 | > 50 | 119 |
| 2 | LY2157299 | 5.16 ± 0.887 | > 50 | > 50 | 9.68 |
| 3 | LY364947 | 4.01 ± 0.562 | > 50 | > 50 | 12.4 |
| 4 | GW788388 | 6.20 ± 0.945 | > 50 | > 50 | 8.05 |
| 5 | EW7197 | 26.0 ± 3.73 | > 50 | 37.8 ± 3.78 | 1.45 |
| 6 | SB431542 | 24.7 ± 2.46 | > 50 | 28.8 ± 2.13 | 1.16 |
| 7 | SB525334 | 15.2 ± 1.68 | > 50 | > 50 | 3.29 |
| 8 | Lurasidone | 1.31 ± 0.133 | 1.81 ± 0.112 | > 50 | 38.0 |
| 9 | Perospirone | 3.73 ± 0.290 | 3.39 ± 0.300 | 22.0 ± 1.30 | 5.92 |
| 10 | Ziprasidone | 0.772 ± 0.0556 | 1.13 ± 0.268 | 6.16 ± 0.498 | 7.98 |
| 11 | Iloperidone | 8.49 ± 1.15 | > 50 | > 50 | 5.89 |
| 12 | Risperidone | > 50 | > 50 | > 50 | 1.00 |
| 13 | FPL64176 | < 0.0976 | 0.270 ± 0.0400 | 46.6 ± 2.50 | 478 |
| 14 | (S)-Bay K8644 | 19.8 ± 1.68 | 25.8 ± 2.80 | > 50 | 2.52 |
| 15 | (R/S)-Bay K8644 | 7.96 ± 0.568 | > 50 | 17.0 ± 1.30 | 2.14 |
| 16 | GP1a | 1.29 ± 0.0803 | 1.53 ± 0.156 | 4.96 ± 0.704 | 3.84 |
| 17 | HU308 | 5.50 ± 0.475 | 12.5 ± 1.28 | 11.8 ± 0.692 | 2.16 |
| 18 | Antrafenine | 1.88 ± 0.130 | > 50 | > 50 | 26.4 |
| 19 | Buclizine | 2.50 ± 0.225 | > 50 | 16.7 ± 2.12 | 6.67 |
| 20 | Calpeptin | 2.79 ± 0.384 | > 50 | 48.4 ± 2.47 | 17.3 |
| 21 | Clomifene | 1.53 ± 0.0573 | 5.95 ± 2.62 | 5.95 ± 3.36 | 3.89 |
| 22 | FGIN-1-27 | 3.13 ± 0.545 | 3.89 ± 0.895 | 47.9 ± 5.67 | 15.3 |
| 23 | GR46611 | 29.7 ± 1.06 | > 50 | > 50 | 1.68 |
| 24 | Guanabenz | 8.68 ± 1.19 | 12.4 ± 3.39 | 48.7 ± 2.57 | 5.61 |
| 25 | Lucanthone | 1.99 ± 0.136 | 5.92 ± 0.493 | 5.92 ± 0.390 | 2.97 |
| 26 | Luliconazole | < 0.0976 | < 0.0976 | 49.2 ± 2.51 | 504 |
| 27 | Pimecrolimus | 1.92 ± 0.147 | 1.66 ± 0.176 | 1.66 ± 0.176 | 0.86 |
| 28 | Retapamulin | 35.1 ± 3.36 | > 50 | > 50 | 1.42 |
| 29 | SR59230A | 4.40 ± 0.269 | 4.68 ± 0.752 | 12.7 ± 0.894 | 2.90 |
| 30 | Tolvaptan | 47.2 ± 2.08 | > 50 | > 50 | 1.06 |
| Ref | Benznidazole | < 0.781 | 4.97 ± 0.698 | > 400 | > 511 |

**Supplementary Table S8.** IC_50_ values of PAR, IR, CC_50_ values in U2OS cells, and SI for 30 selected hits tested in *T. cruzi* Dm28c intracellular amastigote assays after 120 hours treatment.

| **No.** | **Compound** | **PAR (μM)** | **IR (μM)** | **CC_50_ (μM)** | **SI (CC_50_/PAR)** |
| --- | --- | --- | --- | --- | --- |
| 1 | LY2109761 | 0.369 ± 0.0701 | 1.01 ± 0.431 | 47.8 ± 3.84 | 129 |
| 2 | LY2157299 | 4.63 ± 0.632 | 10.4 ± 0.905 | > 50 | 10.7 |
| 3 | LY364947 | 4.17 ± 0.296 | 8.31 ± 0.433 | > 50 | 11.9 |
| 4 | GW788388 | 4.62 ± 0.605 | > 50 | > 50 | 10.8 |
| 5 | EW7197 | 25.4 ± 3.05 | > 50 | 34.4 ± 1.91 | 1.35 |
| 6 | SB431542 | 28.4 ± 4.95 | 47.5 ± 7.77 | > 50 | 1.76 |
| 7 | SB525334 | 6.42 ± 1.90 | > 50 | > 50 | 7.78 |
| 8 | Lurasidone | 1.42 ± 0.104 | 1.78 ± 0.0918 | > 50 | 35.0 |
| 9 | Perospirone | 3.17 ± 0.298 | 3.71 ± 0.439 | 22.9 ± 2.51 | 7.23 |
| 10 | Ziprasidone | 0.909 ± 0.0933 | 1.20 ± 0.198 | 18.0 ± 1.85 | 19.8 |
| 11 | Iloperidone | 11.0 ± 0.979 | > 50 | > 50 | 4.54 |
| 12 | Risperidone | 41.4 ± 2.20 | > 50 | > 50 | 1.21 |
| 13 | FPL64176 | 0.210 ± 0.0249 | 0.290 ± 0.0246 | 34.9 ± 2.57 | 165 |
| 14 | (S)-Bay K8644 | 18.4 ± 3.71 | 22.3 ± 0.421 | > 50 | 2.70 |
| 15 | (R/S)-Bay K8644 | 7.81 ± 0.562 | > 50 | 16.9 ± 2.02 | 2.17 |
| 16 | GP1a | 1.41 ± 0.139 | 2.22 ± 0.845 | 5.76 ± 0.736 | 4.07 |
| 17 | HU308 | 6.44 ± 0.898 | 13.6 ± 0.908 | 12.3 ± 0.651 | 1.92 |
| 18 | Antrafenine | 1.94 ± 0.172 | > 50 | > 50 | 25.6 |
| 19 | Buclizine | 2.50 ± 0.148 | > 50 | 14.5 ± 2.38 | 5.82 |
| 20 | Calpeptin | 4.17 ± 0.492 | 13.3 ± 3.19 | 49.5 ± 6.20 | 11.8 |
| 21 | Clomifene | 1.29 ± 0.0733 | 2.74 ± 0.441 | 3.02 ± 0.218 | 2.34 |
| 22 | FGIN-1-27 | 3.19 ± 1.13 | 3.74 ± 1.31 | > 50 | 15.6 |
| 23 | GR46611 | 25.5 ± 1.26 | > 50 | > 50 | 1.95 |
| 24 | Guanabenz | 7.70 ± 1.49 | 14.0 ± 1.15 | > 50 | 6.49 |
| 25 | Lucanthone | 2.24 ± 0.0944 | 4.78 ± 0.105 | 1.98 ± 0.134 | 0.89 |
| 26 | Luliconazole | < 0.0976 | < 0.0976 | 48.0 ± 2.47 | 493 |
| 27 | Pimecrolimus | 1.44 ± 0.0812 | 1.97 ± 0.126 | 9.01 ± 2.31 | 6.22 |
| 28 | Retapamulin | 28.7 ± 3.30 | 44.3 ± 4.11 | > 50 | 1.74 |
| 29 | SR59230A | 4.72 ± 0.362 | 1.84 ± 0.403 | 12.6 ± 1.51 | 2.67 |
| 30 | Tolvaptan | 49.2 ± 2.92 | > 50 | > 50 | 1.02 |
| Ref | Benznidazole | 0.200 ± 0.00368 | 1.56 ± 0.0830 | > 400 | 1999 |

**Supplementary Table S9.** IC_50_ values for 30 selected hits tested against *T. cruzi* Y epimastigotes for 72 hours and trypomastigotes for 24 hours.

| **No.** | **Compound** | **EPI, 72h (μM)** | **TCT, 24h(μM)** |
| --- | --- | --- | --- |
| 1 | LY2109761 | > 50 | > 100 |
| 2 | LY2157299 | > 50 | > 100 |
| 3 | LY364947 | > 50 | > 100 |
| 4 | GW788388 | > 50 | > 100 |
| 5 | EW7197 | 27.1 ± 3.42 | 46.6 ± 1.87 |
| 6 | SB431542 | > 50 | 46.9 ± 1.88 |
| 7 | SB525334 | 12.1 ± 0.974 | > 100 |
| 8 | Lurasidone | 15.1 ± 2.51 | 7.43 ± 0.747 |
| 9 | Perospirone | 18.6 ± 1.03 | 6.59 ± 0.181 |
| 10 | Ziprasidone | > 50 | > 100 |
| 11 | Iloperidone | > 50 | 38.1 ± 1.51 |
| 12 | Risperidone | > 50 | > 100 |
| 13 | FPL64176 | 2.37 ± 0.126 | 52.7 ± 3.93 |
| 14 | (S)-Bay K8644 | > 50 | 53.8 ± 0.887 |
| 15 | (R/S)-Bay K8644 | > 50 | 55.9 ± 1.40 |
| 16 | GP1a | 14.6 ± 1.03 | 13.3 ± 0.567 |
| 17 | HU308 | > 50 | 31.4 ± 2.41 |
| 18 | Antrafenine | > 50 | > 100 |
| 19 | Buclizine | > 50 | > 100 |
| 20 | Calpeptin | 35.0 ± 1.30 | 12.5 ± 2.17 |
| 21 | Clomifene | 32.1 ± 0.930 | 9.16 ± 0.394 |
| 22 | FGIN-1-27 | > 50 | 42.3 ± 3.10 |
| 23 | GR46611 | > 50 | 27.3 ± 0.925 |
| 24 | Guanabenz | 45.1 ± 1.86 | 42.9 ± 2.62 |
| 25 | Lucanthone | 17.6 ± 0.648 | 11.9 ± 1.68 |
| 26 | Luliconazole | > 50 | 45.8 ± 1.49 |
| 27 | Pimecrolimus | > 50 | 7.32 ± 0.738 |
| 28 | Retapamulin | > 50 | 26.9 ± 1.11 |
| 29 | SR59230A | 44.8 ± 1.74 | 10.5 ± 0.391 |
| 30 | Tolvaptan | 47.7 ± 1.04 | 67.7 ± 3.98 |
| Ref | Benznidazole | 18.7 ± 1.51 | 10.3 ± 1.35 |

**Supplementary Table S10.** IC_50_ values for 30 selected hits tested against *T. cruzi* Dm28c epimastigotes for 72 hours and trypomastigotes for 24 hours.

| **No.** | **Compound** | **EPI, 72h (μM)** | **TCT, 24h(μM)** |
| --- | --- | --- | --- |
| 1 | LY2109761 | 2.98 ± 0.521 | 11.4 ± 5.12 |
| 2 | LY2157299 | > 100 | > 100 |
| 3 | LY364947 | 73.1 ± 9.73 | > 100 |
| 4 | GW788388 | > 100 | 60.6 ± 4.44 |
| 5 | EW7197 | 51.2 ± 1.97 | 47.8 ± 11.0 |
| 6 | SB431542 | 63.3 ± 7.14 | 35.8 ± 4.31 |
| 7 | SB525334 | > 100 | > 100 |
| 8 | Lurasidone | 5.23 ± 0.144 | 4.76 ± 0.523 |
| 9 | Perospirone | 14.3 ± 0.651 | 6.45 ± 0.317 |
| 10 | Ziprasidone | > 100 | > 100 |
| 11 | Iloperidone | 79.9 ± 3.37 | 41.2 ± 2.80 |
| 12 | Risperidone | > 100 | > 100 |
| 13 | FPL64176 | < 0.781 | 50.2 ± 5.23 |
| 14 | (S)-Bay K8644 | 52.7 ± 1.72 | 48.4 ± 9.28 |
| 15 | (R/S)-Bay K8644 | 97.4 ± 1.78 | 51.8 ± 7.03 |
| 16 | GP1a | 10.8 ± 1.25 | 5.87 ± 1.27 |
| 17 | HU308 | > 100 | 8.55 ± 0.448 |
| 18 | Antrafenine | > 100 | > 100 |
| 19 | Buclizine | 51.9 ± 1.31 | 9.07 ± 0.657 |
| 20 | Calpeptin | 4.93 ± 0.499 | < 0.781 |
| 21 | Clomifene | 20.3 ± 0.434 | 4.00 ± 0.356 |
| 22 | FGIN-1-27 | > 100 | 7.31 ± 0.752 |
| 23 | GR46611 | > 100 | 25.2 ± 2.40 |
| 24 | Guanabenz | > 100 | 24.2 ± 3.18 |
| 25 | Lucanthone | 4.64 ± 0.330 | 6.25 ± 3.45 |
| 26 | Luliconazole | 25.2 ± 5.11 | 19.4 ± 1.85 |
| 27 | Pimecrolimus | > 100 | 1.65 ± 0.315 |
| 28 | Retapamulin | 80.1 ± 8.98 | 24.3 ± 2.79 |
| 29 | SR59230A | 21.1 ± 2.66 | 6.01 ± 0.739 |
| 30 | Tolvaptan | 24.8 ± 4.39 | 29.9 ± 5.57 |
| Ref | Benznidazole | 11.6 ± 0.590 | 24.8 ± 1.10 |

**Supplementary Table S11.** IC_50_ values of reference compounds tested against *T. cruzi* Y trypomastigotes for 24 hours.

| **No.** | **Compound** | **TCT, 24h(μM)** |
| --- | --- | --- |
| 1 | Benznidazole | 10.3 ± 1.35 |
| 2 | Nifurtimox | 25.0 ± 3.79 |
| 3 | Amphotericin B | 0.361 ± 0.0657 |
| 4 | Pentamidine | 9.20 ± 0.459 |
| 5 | Clofazimine | 50.0 ± 4.25 |
| 6 | Sitamaquine | 10.3 ± 0.961 |
| 7 | Tafenoquine | 26.3 ± 2.98 |
| 8 | Aminopyrazole  2491044 | 50.0 ± 3.59 |
| 9 | Aminopyrazole  3095561 | 49.9 ± 3.14 |
